# A Comparison of Structural and Evolutionary Attributes of *Escherichia coli* and *Thermus thermophilus* Small Ribosomal Subunits: Signatures of Thermal Adaptation

**DOI:** 10.1371/journal.pone.0069898

**Published:** 2013-08-05

**Authors:** Saurav Mallik, Sudip Kundu

**Affiliations:** Department of Biophysics, Molecular Biology and Bioinformatics, University of Calcutta, Kolkata, India; University of Houston, United States of America

## Abstract

Here we compare the structural and evolutionary attributes of *Thermus thermophilus* and *Escherichia coli* small ribosomal subunits (SSU). Our results indicate that with few exceptions, thermophilic 16S ribosomal RNA (16S rRNA) is densely packed compared to that of mesophilic at most of the analogous spatial regions. In addition, we have located species-specific cavity clusters (SSCCs) in both species. *E. coli* SSCCs are numerous and larger compared to *T. thermophilus* SSCCs, which again indicates densely packed thermophilic 16S rRNA. Thermophilic ribosomal proteins (r-proteins) have longer disordered regions than their mesophilic homologs and they experience larger disorder-to-order transitions during SSU-assembly. This is reflected in the predicted higher conformational changes of thermophilic r-proteins compared to their mesophilic homologs during SSU-assembly. This high conformational change of thermophilic r-proteins may help them to associate with the 16S ribosomal RNA with high complementary interfaces, larger interface areas, and denser molecular contacts, compared to those of mesophilic. Thus, thermophilic protein-rRNA interfaces are tightly associated with 16S rRNA than their mesophilic homologs. Densely packed 16S rRNA interior and tight protein-rRNA binding of *T. thermophilus* (compared to those of *E. coli*) are likely the signatures of its thermal adaptation. We have found a linear correlation between the free energy of protein-RNA interface formation, interface size, and square of conformational changes, which is followed in both prokaryotic and eukaryotic SSU. Disorder is associated with high protein-RNA interface polarity. We have found an evolutionary tendency to maintain high polarity (thereby disorder) at protein-rRNA interfaces, than that at rest of the protein structures. However, some proteins exhibit exceptions to this general trend.

## Introduction

The modern ribosome is a sophisticated ribonucleoprotein complex having a large subunit (LSU) and a small subunit (SSU). The bacterial SSU typically contains a 16S ribosomal RNA (rRNA) and 24 ribosomal proteins (r-protein) attached to it, whereas the LSU contains a 23S rRNA, 5S rRNA and 34 proteins [1]. In the Universal Phylogenetic Tree (UPT), 34 ribosomal proteins (15 of SSU and 19 of LSU) are observed to have homologs in all three phylogenetic domains [2]. The prokaryotic ribosome (SSU and LSU) structures have been resolved at molecular level by high-resolution X-ray crystallography [3]–[10]. These works provide a deep insight into the structure of ribosomal RNAs and proteins and their molecular interactions. The association pathways of both the subunits have also been analyzed in detail [11]–[14]. It was shown that the rRNA does not fold into its functional state without the presence of r-proteins [15], [16]. Thus, general purpose of the r-proteins is to assist rRNA folding and provide structural stability to the folded rRNAs [4]. Now, apart from this general strategy, if some structural/evolutionary attributes are observed to be significantly different for two species at the same domain of life, but habituated at different environmental conditions (one mesophilic and another thermophilic), then that difference might entail an adaptation strategy with the environment.

Every organism is adapted according to its habitat environment. The study of molecular strategies to adapt with the environment is a very interesting scientific field to work on. The thermal adaptation has long been at the center of such studies. Researchers have analyzed these signatures in genome [17], [18], transcriptome [19], and proteome level [19]–[26]. Proteome level studies confirm that a significant reduction in the frequency of the thermolabile amino acids (histidine, glutamine, and threonine) and an increment in the frequency of charged residues (arginine, lysine etc) is a common signature of thermal adaptation [25], [26]. At the structure level, genomic/transcriptome/proteome level natural selections are directed towards the generation of thermostable biomolecules. Densely packed interior is a common attribute of the thermophilic biomolecules, although there are exceptions [20]–[22]. Researchers of this field have mostly worked on monomeric proteins to study the effects of thermal adaptation. Thus, a detailed study on the nature of thermal adaptation at the biomolecular interfaces is still unavailable. However, it is known that thermophilic and hyperthermophilic proteins have reduced disordered regions compared to mesophilic; proteins related to translation and ribosome biogenesis show an exception to this trend [27]. In this current work, we have established that this disorder is preferably maintained at the interfaces of the r-proteins. At thermophilic conditions, the disorder-to-order transitions due to protein-rRNA interactions generate a strong binding between protein and rRNA constituents of SSU.

In this current work, we have calculated the different structural and evolutionary attributes of *Escherichia coli* and *Thermus thermophilus* SSU and have made pairwise comparisons. Since SSU is a ribonucleoprotein oligomer, we have conducted our investigation at two levels: (1) what are the signatures of thermal adaptation at the 16S rRNA interior, (2) how the protein-rRNA interactions result towards generation of highly stable SSU structures in thermophilic bacteria.

## Results/Discussions

### Cavity Analysis of *E. coli* and *T. thermophilus* SSU

The SSU is a large ribonucleoprotein complex containing a large 16S rRNA and about 24 proteins. We begin our work by comparing the distribution of cavities in the *E. coli* and *T. thermophilus* SSUs. Cavities are located both within the interior (cavities) and on the surface (pockets/clefts) of biomolecules [28], [29]. The cavities in the interior of biomolecules indicate loose internal packing [30] and their presence can even reduce the structural stability [31]. On the other hand, the presences of cavities at the oligomeric interfaces reduce the interface complementarity [29]. In this current work, we have compared the distribution of cavities at rRNA-interiors (RI cavities) and at protein-rRNA interfaces (PR cavities) of thermophilic and mesophilic SSUs. PR cavities are also divided into two groups: cavities contributed by one single protein and 16S rRNA (SPR) and those contributed by multiple proteins and 16S rRNA (MPR) at the interface.

#### Cavities in 16S rRNA

**Figure 1:**
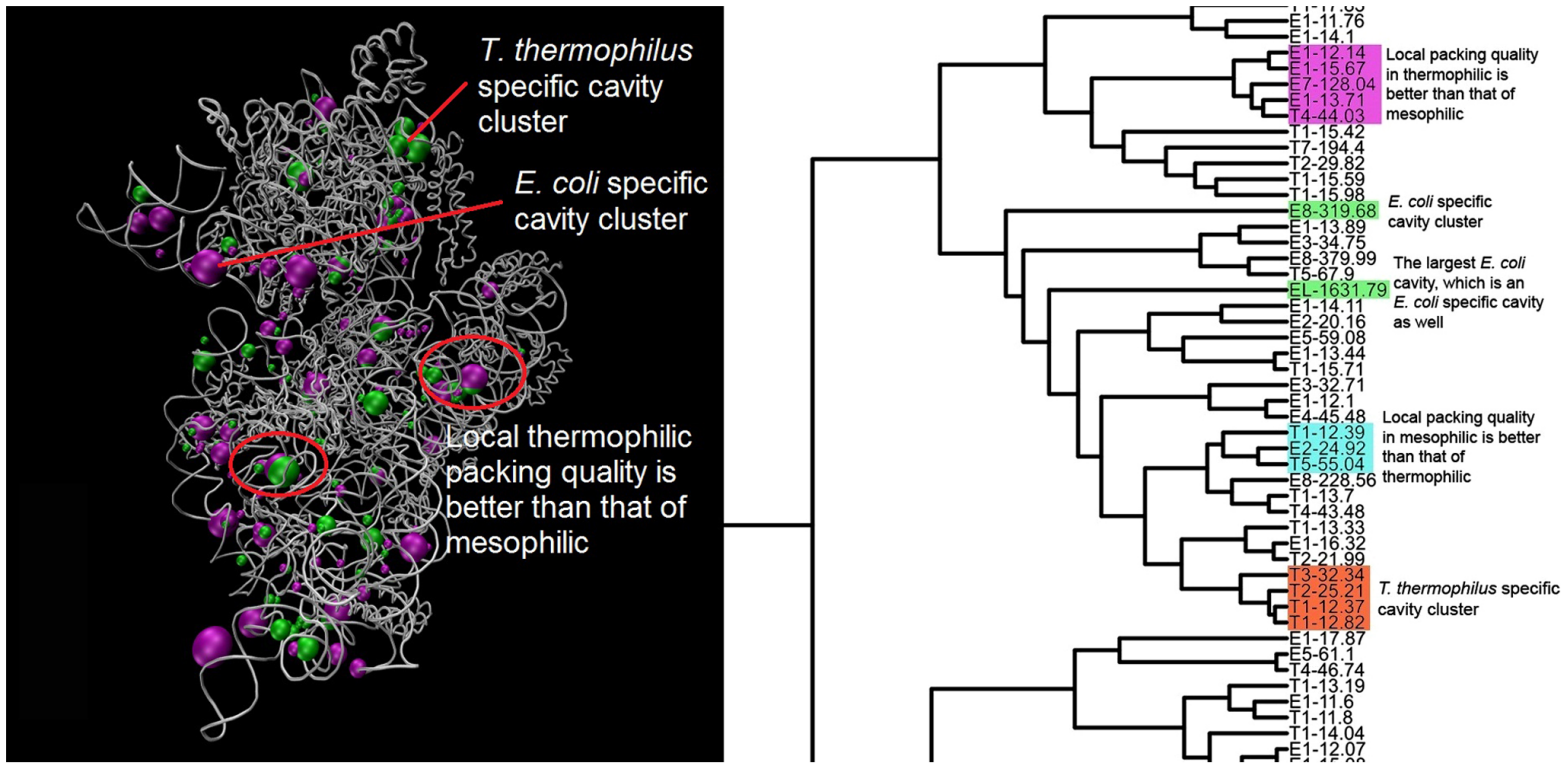
The position of cavities in 16S rRNA and the cavity clustering dendogram is shown. (Left) The cavities in structurally aligned 16S rRNAs of *E. coli* (white tube view) and *T. thermophilus* (not shown) are presented. Cavities are presented as magenta (*E. coli*) and green (*T. thermophilus*) spheres. SSCC and non-SSCC cavities are highlighted accordingly. (Right) A part of the dendogram of positional clustering of cavities is presented in linear mode. Notations used in the dendogram: Cavities within 16S rRNAs (RI cavities) were classified into eight groups according to their size: 10–20 Å^3^ – T1 and E1, 20–30 Å^3^ – T2 and E2; 30–40 Å^3^ – T3 and E3, 40–50 Å^3^ – T4 and E4, 50–70 Å^3^ – T5 and E5, 70–100 Å^3^ – T6 and E6, 100–200 Å^3^ – T7 and E7 and > 200 Å^3^ – T8 and E8. Along with each cavity, its volume is also shown.

Here, we have aligned the structures of the 16S rRNAs (see File S2) of the *E. coli* and *T. thermophilus,* and then have identified their cavities. Our results show that the largest cavity in *T. thermophilus* 16S rRNA (structure PDB-id: 1FJG) contains 289.49 Å^3^ volume; whereas the largest *E. coli* 16S rRNA (structure PDB-id: 2AVY) cavity contains 1631.79 Å^3^ volume of space. The average sphericity of the 16S rRNA interior cavities is 1.12 (SD = 0.71). Therefore, if we approximate them as spheres, it would not be associated with much computational error. We have calculated the central points of each of the cavities and have performed a positional clustering (UPGMA method) taking all the thermophilic and mesophilic cavities together. Here, we cluster the cavities located at the analogous spatial regions of two aligned structures into the same group. Thus, this clustering enables us to compare the local packing quality (determined by the abundance and size of cavities) of 16S rRNA at every analogous spatial region of the two species. The 16S rRNA has four domains, identified by its secondary structure: 5′-domain, central domain, 3′-major domain and 3′-minor domain [32]. The nexus file of the cavity clustering dendogram is provided as File S3. A part of the dendogram is shown in Figure-1 (right). It shows that cavities located at same 16S rRNA domains are generally clustered into specific branches. However, some inter-domain cavities are also observed in these domain-specific branches.

The most useful application of this clustering is that it allows us to compare the distribution of the RI cavities throughout the 16S rRNA structure of the two species. When we compare the cavity distribution at different SSU structures of the same species, we see that 82% of *E. coli* and 88% of *T. thermophilus* cavities having volume ≥ 40 Å^3^ are ‘universally conserved’ across structures. We consider a cavity to be ‘conserved’ across structures, if at the analogous spatial regions each structure contains a cavity of comparable size. However, position and size of smaller cavities (10–20 Å^3^) vary irregularly across structures. The cavity clustering dendogram clearly shows us that the spatial distribution of 16S rRNA cavities is not identical in the two species. Instead, there are some **‘Species-Specific Cavity Clusters’ (SSCC)**. These are analogous spatial regions of thermophilic and mesophilic 16S rRNAs, where cavities are observed for only one species; but that space is completely devoid of cavities in the other species (see Figure-1 (left)). We have identified that a SSCC might be one single cavity or it might be a dense cluster of many small cavities. Now, we define a term cluster volume (CV), which is the sum of the volumes of all members of a SSCC. This parameter defines the spatial occupancy of a SSCC, irrespective of the number of its members. According to their CV values, we have classified the SSCCs into three groups: small (CV<150 Å^3^), medium (150Å^3^<CV<300Å^3^) and large (CV>300Å^3^). The reason we opted 150Å^3^ and 300Å^3^ as our marker of SSCC size is that, the former is approximately the van der Waals volume of a uracil/cytosine nucleotide and the later is that of an adenine/guanine. We have observed seventeen SSCCs in the *E. coli* 16S rRNA (five large, seven medium and five small) and ten SSCCs (two large, five medium and three small) in that of *T. thermophilus*.

Next, we have tried to explore if the appearance of large and medium SSCCs are specific to certain domains of 16S rRNA. We find three medium SSCCs in the central domain of mesophilic 16S rRNA, while thermophilic 16S rRNA has only one medium SSCC in the same domain. Similarly, four large and two medium SSCCs are located in 5′-domain of mesophilic 16S rRNA, whereas the thermophilic 5′-domain contains only one large and three medium SSCC. In 3′-major domain, mesophilic 16S rRNA contains one large and two medium SSCC, whereas thermophilic 16S rRNA has only one medium SSCC in the same domain. One large thermophilic SSCC lies at the interface of 5′- and 3′-major domain. Thus, there is no central tendency of SSCC appearance towards any particular domain of 16S rRNA. Thus, we observe the presence of larger and medium SSCCs in mesophilic SSU compared to the thermophilic. It is known that densely packed biomolecular interior is a general signature of thermal adaptation. Thus, we can conclude that the densely packed interior of thermophilic 16S rRNA (compared to mesophilic) is merely signature of thermal adaptation.

Next, we are interested about the non-SSCC cavity clusters, which allow us to compare the abundance and size of cavities (therefore, the packing quality) in the analogous spatial regions of *T. thermophilus* and *E. coli* 16S rRNA structures, where cavities are present in both the species. We have identified 45 non-SSCC cavity clusters. To compare the packing qualities of the two species directly, we measure the CV for each species (CV(coli) and CV(thermo)) in each cluster. Now, for any non-SSCC cavity cluster, the CV(thermo)/CV(coli) ratio being less then unity, would indicate that at the analogous spatial region, thermophilic 16S rRNA is densely packed compared to the mesophilic 16S rRNA. We have identified that 67% of the non-SSCC cavity clusters show CV(thermo)/CV(coli) ratio less than unity, where, 33% clusters exhibit CV(thermo)/CV(coli) ratio >1. Thus, the results indicate that thermophilic 16S rRNA is more densely packed than that of mesophilic in most of the analogous spatial regions. The domain specific distribution of cavity clusters having the values of the ratio >1 is given below. Four of such clusters are located in 5′-Domain, which is the largest domain of 16S rRNA and 10 clusters are located at the central and 3′-Major Domain. These two domains mainly bind with the 50S subunit during translation [20].The (CV(thermo)/CV(coli) ratios are presented in Table S1. Position of cavity clusters having CV(thermo)/CV(coli) ratio>1 are presented in Figure S1.

Thus, the cavity distribution at *T. thermophilus* and *E. coli* 16S rRNA interiors shows us two facts: (1) SSCCs are numerous and larger in *E. coli*, (2) CV(thermo)/CV(coli) is less than 1 in 67% of the non-SSCC cavities. Thus, we can conclude that the local packing quality of the thermophilic 16S rRNA interior is better in most of the analogous spatial regions compared to that of thermophilic 16S rRNA. This dense packing is likely to be a signature of thermal adaptation.

#### Protein-rRNA interface complementarity

Next, we investigate the property of cavities at the protein-rRNA interfaces (PR cavities). Cavity index (CI) measures the cavity volume per unit interface area of two biomolecules; the smaller the CI, the more complementary the interface is [29]. We have estimated the CI values of the *E.* coli (CI(coli)) and *T. thermophilus* (CI(thermo)) protein-rRNA interfaces.

The cavity index values show that thermophilic protein-rRNA interfaces are more complementary compared to those of mesophilic. Taking all r-proteins together, we observe that the average value of *T. thermophilus* cavity index (CI(thermo) = 0.26) is lesser than that of *E. coli* (CI(coli) is 0.32). Next, we calculate the cavity index values of individual protein-rRNA interfaces to identify whether this is a general trend for every homologous pair of SSU r-proteins. Both MPR and SPR cavities (see ‘Cavity Analysis of *E. coli* and *T. thermophilus* SSU’ for definition) are detected in protein-rRNA interfaces for both the species. First, we use only SPR cavities to estimate CI values of individual proteins. We observe that 12 of the thermophilic r-proteins have lower CI values (that means higher complementary rRNA interface) compared to their *E. coli* homologs. Thermophilic S6 associates with 16S rRNA with no detectable cavity in its interface (CI(thermo) = zero). This makes rRNA-interface of this protein perfectly complementary, whereas the *E. coli* S6 protein has 0.17 cavity index. S16 protein of *T. thermophilus* also have highly complementary protein-rRNA interface (CI(thermo) = 0.05), compared to its *E. coli* homolog (CI(coli) = 0.34). S17 is another example of this trend (CI(thermo) = 0.21 and CI(coli) = 0.69).

The homologous r-proteins of *T. thermophilus* and *E. coli* are highly similar to each other both in their length and in three-dimensional structures. In the previous section, we show that the larger number of *T. thermophilus* proteins have lower CI values compared to their *E. coli* homologs and hence, the former one has highly complementary interface. So, when *T. thermophilus* proteins bind with 16S rRNA with higher surface complementary compared to their *E. coli* homologs, it should generate larger interface areas and provide the possibility of numerous atomic contacts (discussed later). This would likely generate stable SSU structures to persist at high temperatures. Thus, highly complementary interface of *T. thermophilus* should be an attribute of thermal adaptation.

However, here we see that S4, S5, S7, S8, S15, S19, and S20 proteins are indicating a contradiction to the general trend that thermophilic proteins should have highly complementary rRNA interfaces compared to their mesophilic homologs. This contradiction partially vanishes when we look at MPR cavities. In SSU, many protein-protein interfaces are detected in both species, containing MPR cavities. Hence, the SPR cavities alone cannot provide us the complete scenario about the protein-rRNA interface complementarity. We have detected 16 MPR cavities in *E. coli* SSU; whereas in *T. thermophilus* SSU, 10 MPR cavities are detected. In *T. thermophilus*, 6 out of 21 proteins (S3, S9, S10, S13, S14, S19 and THX) and in *E. coli*, 12 out of 21 proteins (S4, S5, S6, S8, S9, S10, S11, S12, S13, S14, S18 and S19) contribute to MPR cavities. According to the presence of MPR cavities between the neighboring proteins (neighbor in the sense of three-dimensional position), thermophilic SSU proteins are clustered into two groups: S3-S10-S14 (rRNA interface CI of the whole cluster = 0.13) and S9-S13-S19-THX (CI = 0.25). In *E. coli*, we identify four such groups: S4-S5 (CI = 0.14), S6-S11-S18 (CI = 0.41), S8-S12 (CI = 0.08), and S9-S10-S13-S14-S19 (CI = 0.19). We can see there is no common cluster between the two species and so we cannot compare them directly. However, 16 MPR cavities of *E. coli* altogether occupy 3033.45Å^3^ volume of space, while 10 MPR cavities of *T. thermophilus* occupy 2625.33Å^3^ volume. This indicates that *E. coli* protein-rRNA interfaces contain numerous and larger MPR cavities.

We have observed that S4, S5, S7, S8, S15, S19, and S20 proteins of *E. coli* have smaller cavity indices compared to their *T. thermophilus* homologs. Analysis of the MPR cavities shows that S4, S5 and S19 proteins of *E. coli* contribute to MPR cavities in *E. coli* SSU. On the other hand, in *T. thermophilus*, only S19 contributes to MPR cavities. We have already shown that when we consider all proteins together, we observe that r-proteins of thermophilic SSU associate with 16S rRNA with highly complementary interfaces compared to that of mesophilic. Thus, highly complementary protein-rRNA interfaces seem to be another signature of thermal adaptation in SSU. In Table S2, we have presented the CI values for *E. coli* and *T. thermophilus* SSU r-proteins.

### Significance of high Protein-rRNA interface complementarity

#### High Protein-rRNA interface complementarity is associated with larger interface areas

Structural alignments of *T. thermophilus* and *E. coli* SSU r-proteins using the SSM algorithm [33] show that the homologous proteins have very similar structures and they interact with 16S rRNA at analogous sites. However, the protein-rRNA interface complementarities of the two species are significantly different. We are interested to see whether this higher complementarity is associated with the generation of larger protein-rRNA interface areas (which should be correlated with higher structural stability).

We calculate the protein-rRNA interface areas (buried surface area: BSA). We see that with the exception of S4, S11, S14, and S18, rest of the thermophilic r-proteins have larger protein-rRNA interface area compared to their mesophilic homolog. In Table S3, we have presented the BSA data for the two species. Next, we have tried to correlate whether differences in surface complementarity is monotonically correlated with differences in BSA. We have defined an X-factor for the CI and BSA data of the r-proteins as:
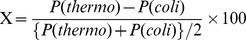
Here, P(thermo) and P(coli) indicates the parameter values of *T. thermophilus* and *E. coli*. Here, the parameter X is defined such that <X> indicates the magnitude of difference between P(thermo) and P(coli) and positive sign of X indicates that corresponding parameter has higher value in *T. thermophilus*, compared to *E. coli* and vice versa. We have estimated X values for both BSA (X_BSA_) and CI (X_CI_) for the r-proteins. The Spearman's rank correlation coefficient between X_BSA_ and X_CI_ is 0.93, which indicates that the relationship between this two variables can be described using some monotonic function. In other words, the lower cavity index of thermophilic r-proteins (compared to mesophilic) usually results in their larger rRNA-interface area, compared to those of mesophilic. However, this exact mathematical correlation between the two parameters is unclear.

#### Interface complementarity and the location of r-proteins in SSU

While analyzing the structural attributes of the homologous SSU r-proteins of *T. thermophilus* and *E. coli*, we observe that 11 out of 19 homologous r-proteins exhibit higher rRNA-interface complementarity in *T. thermophilus* (see Table S2). On the other hand, 15 out of 19 homologous r-proteins bind with 16S rRNA with larger interface areas (see Table S3). A total of eight SSU r-proteins (S2, S3, S6, S9, S12, S13, S16 and S17) exhibit higher BSA and higher rRNA-interface complementarity simultaneously in *T. thermophilus* compared to their mesophilic homologs. The only S4 protein has higher BSA and complementarity in *E. coli*.

The entire SSU can be subdivided into two parts: the ‘head’ and the ‘body’ portions, attached to each other through a narrow ‘neck’ [15]. The ‘head’ portion is constituted by the 3′-Major domain of the 16S rRNA, while the other three domains (5′, Central, and 3′-minor) constitute the ‘body’ portion (see Figure S2). Therefore, the entire 16S rRNA is constituted such that two large masses are joined to each other via the narrow ‘neck’ portion (area of interaction is about 1200Å^2^). Three r-proteins (parts of S2, S3 and S5) interact with this neck region. Out of them, S2 and S3 exhibit higher BSA and higher rRNA-interface complementarity in *T. thermophilus*, while S5 exhibits higher complementarity in *E. coli*, but larger surface area in *T. thermophilus*.

Next, we discuss about eight proteins (S2, S3, S7, S9, S10, S13, S14, and S19) which are found at the ‘head’ portion of SSU. Except S14, each of them has higher rRNA-interface area in *T. thermophilus*. Among rest of the seven proteins, four (S2, S3, S9 and S13) have higher interface complementarity in *T. thermophilus*, two (S7 and S19) have higher complementarity in *E. coli*. Only in case of protein S10, we see interface complementarities are approximately the same in both the species.

Proteins exhibiting higher interface complementarity in mesophilic are mostly (except S7 and S19) clustered at the ‘body’ portion of SSU (S4, S5, S8, S15, and S20). Except S20, all the others interact with both the Central and 5′-domains (S20 interacts with 5′- and 3′-minor domains). At the ‘body’ portion, we have located five proteins (S6, S11, S16, S17 and S18) with higher interface complementarity in *T. thermophilus*. The proteins S6, S11 and S18 are clustered very close to each other; and all of them interact only with the Central domain of 16S rRNA (and between themselves as well). S16 and S17 simultaneously exhibit higher BSA and complementarity in T. thermophilic; and they interact with both the Central and 5′-domains. The protein S6 also having higher BSA and complementarity in *T. thermophilus* bind with the central domain. Protein S12 is located at the 30S–50S interface side of the SSU. This protein has very comparable interface complementarity in both thermophilic and mesophilic.

### Protein conformational changes during SSU association

We have already established that larger number of SSU r-proteins of thermophilic bacteria associate with 16S rRNA with larger interface areas compared to those of mesophilic. Now, we have predicted the conformational changes of the r-proteins during SSU association to investigate whether the generation of larger interface areas is correlated with higher conformational changes of thermophilic r-proteins.

Most of the ribosomal proteins have several extended domains buried in RNA; being isolated from the subunit, they do not contain these extensions [4]. Due to this problem, only a few crystal structures of r-proteins in their free cytosolic state are available in PDB (see File S1 for our dataset). Thus, we have used the algorithm of Marsh and Teichmann [34], which was primarily suggested to predict the conformational changes of protein monomers when they oligomerize. This algorithm (see File S2) was designed for the cases when only the complexed structures (not the uncomplexed structures) are available in database. We have utilized this concept to predict the conformational changes (expressed as Root Mean Square Distances (RMSD) between the complexed and uncomplexed states) of SSU r-proteins as they associate with 16S rRNA; in addition, this algorithm can provide prediction of the structural flexibility (expressed as ASA(rel)) of protein monomers at their uncomplexed state. The results are summarized in Table 1. The predicted RMSD values vary in a wide range for both the species.

**Table 1:** The ASA(rel) values (expressing the structural flexibility of proteins in their uncomplexed state) and predicted conformational changes (RMSD) of small subunit ribosomal proteins due to association with 16S rRNA are enlisted here.

Ribosomal proteins	ASA(rel) values for *T. thermophilus*	ASA(rel) values for *E. coli*	Predicted RMSD values for *T. thermophilus* (Å)	Predicted RMSD values for *E. coli* (Å)
S2	1.10 (0.04)	1.12 (0.05)	2.98 (0.92)	2.63 (0.70)
S3	1.20 (0.03)	1.22 (0.05)	5.61 (2.06)	5.01 (0.91)
S4	1.16 (0.02)	1.19 (0.05)	4.71 (1.78)	3.66 (0.49)
S5	1.12 (0.03)	1.12 (0.03)	2.99 (0.67)	2.93 (0.64)
S6	1.03 (0.03)	1.16 (0.05)	3.89 (1.40)	1.67 (0.34)
S7	1.22 (0.05)	1.27 (0.04)	7.69 (2.14)	5.70 (1.54)
S8	1.10 (0.02)	1.12 (0.04)	3.07 (0.87)	2.59 (0.26)
S9	1.21 (0.02)	1.24 (0.05)	6.56 (2.42)	5.00 (0.55)
S10	1.27 (0.02)	1.29 (0.05)	9.12 (2.71)	7.57 (1.22)
S11	1.20 (0.02)	1.22 (0.03)	5.56 (1.06)	4.88 (0.69)
S12	1.33 (0.03)	1.41 (0.05)	18.76 (5.47)	11.14 (1.92)
S13	1.28 (0.03)	1.36 (0.06)	14.25 (5.67)	8.10 (1.36)
S14	1.40 (0.03)	1.41 (0.04)	19.19 (5.32)	17.50 (3.45)
S15	1.17 (0.01)	1.22 (0.06)	5.78 (2.23)	3.90 (0.34)
S16	1.13 (0.01)	1.08 (0.04)	2.25 (0.57)	3.04 (0.28)
S17	1.21 (0.02)	1.30 (0.05)	9.79 (3.32)	5.08 (0.57)
S18	1.14 (0.03)	1.20 (0.04)	5.04 (1.13)	3.31 (0.65)
S19	1.20 (0.02)	1.25 (0.05)	6.83 (2.06)	4.80 (0.62)
S20	1.23 (0.01)	1.23 (0.22)	8.94 (5.16)	5.72 (0.29)
S21	-	1.18 (0.04)	-	32.26 (11.32)
THX	1.49 (0.06)	-	4.30 (1.06)	-

The ASA(rel)>1.2 indicates the protein is structurally flexible and ASA(rel)>1.4 indicates intrinsic disorder of corresponding proteins. We can observe that S12 and S14 are intrinsically disordered, and S13 has a very high structural flexibility. On the other hand, S2, S6, S16 are structurally rigid proteins. Numbers in the parenthesis indicate corresponding standard deviation values.

Following the work of Marsh and Teichmann, we cluster the r-proteins (UPGMA method) into four groups in increasing structural flexibility order: (Group-A) S2, S5, S6, S8, S16, (Group-B) S3, S4, S7, S9, S11, S15, S19; (Group-C) S10, S17, S18, S20; (Group-D) S12, S13, S14. Group-A and B proteins are found on 16S rRNA surface. They have small RNA buried extensions, rigid structures and undergo small conformational changes upon interaction with 16S rRNA (average RMSD on both species are 2.80Å and 5.14Å; respectively). Group-C proteins have intermediate structural flexibility, comparatively longer RNA buried extensions; and they undergo intermediate structural changes (average RMSD = 7.45Å). Group-D proteins have long RNA buried extensions and highly flexible structures which experience large structural changes (average RMSD = 14.82Å). We found that Group-A and B and Group-B and C are significantly different from each other by U-test (p<0.01). Group-D is marginally different from every other group (0.01<p<0.05). Group-A and C are also marginally different (0.01<p<0.05). During the SSU assembly, the 16 rRNA is largely folded (post-transcription) and has most secondary structures in place. The proteins essentially hold these pieces together in a certain orientation. This is reflected in the Nomura Assembly Map [11] and has been confirmed by the recent studies as well [12]–[14]. Now, the protein-rRNA association would likely occur through some structural changes of the r-proteins (compared to their uncomplexed state). Some r-proteins exhibit long extensions at their complexed state (assembled to SSU), which pierce into the rRNA-interior (Group-C and D), while the others (Group-A and B) bind at the rRNA-surface, having small or no extensions at all [15]. Proteins exhibiting long rRNA-buried extensions (in their complexed state) would likely experience large conformational changes (with respect to their uncomplexed states), compared to proteins binding only at rRNA-surface. Therefore, the nature of interaction with the rRNA determines the amount of structural changes of the r-proteins (however, assembly timing is not likely correlated with the amount of structural changes).

Marsh and Teichmann's algorithm shows that (ASA(rel) values) thermophilic and mesophilic r-proteins have almost similar structural rigidity at their free cytosolic states, with a slight tendency that thermophilic proteins are more rigid. However, except S16, every other thermophilic protein undergoes higher conformational changes (Table 1, RMSD data) compared to their mesophilic homologs. The larger structural changes of thermophilic r-proteins likely enable them to interact with 16S rRNA with larger interface areas than their mesophilic homologs. Following the method described in ‘High Protein-rRNA interface complementarity is associated with larger interface areas’, we have estimated the X_RMSD_ values in this case and have correlated them with X_BSA_ data. We have found that Spearman's rank correlation coefficient calculated between X_BSA_ and X_RMSD_ is 0.56 (Linear correlation coefficient is 0.54). Thus, it seems that BSA is not the only factor determining the conformational changes of r-proteins.

### Protein conformational changes follow a general trend for both the species

Next, we try to understand whether the physical properties of protein-rRNA interfaces of the two species have any effect on their predicted conformational changes. Earlier works [35], [36] have shown that in case of protein-protein interactions, the conformational changes of the monomers depend on their interface areas. Here, we have tested whether there is any relationship between conformational changes, and the strength of protein-rRNA interaction, represented by solvent free energy (ΔG) of association and interface size (Buried Surface Area: BSA). We have calculated the BSA and ΔG values of rRNA-association for each homologous r-protein of *T. thermophilus* and *E. coli* (see File S2). A careful analysis shows that in ribosomal protein-RNA interactions (using data for the three species together), there exists a very low value of linear correlation coefficient (r-value = 0.35, R-square = 0.13) between conformational change (RMSD) and BSA. We also observe a poor linear fitting of RMSD and ΔG of association (r-value = 0.27, R-square = 0.09). Interestingly, the three parameters, all-together, correlate very well with each other in a linear equation (r-value = 0.95 and R-square = 0.88), defining a plane surface in a three-dimensional space. The linear equation is shown in the following:
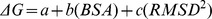
Here a, b and c are the fitting constants. A 3D surface plot of this fitting is presented in Figure S3. This very equation is observed in both the bacterial species (*T. thermophilus* and *E. coli*) and is followed in a eukaryotic ribosome (*Tetrahymena thermophila*) as well. However, the values of the coefficients, a, b and c are species specific (Table S4). In Table S5, the ΔG values of r-proteins are presented. It shows that all mesophilic and thermophilic r-proteins experience conformational changes upon SSU-association following the same equation. Hence, the amount of conformational changes depends on the interface size (determined by complementarity) and ΔG of protein-rRNA association. ΔG of association again depends on interface size, electrostatic interactions, and molecular contacts between the interacting partners [37], [38]. Highly complementary thermophilic protein-rRNA interfaces (CI = 0.26) compared to mesophilic (CI = 0.32) likely have a positive effect on the surface density of molecular contacts. We have identified that thermophilic protein-rRNA interfaces are enriched in molecular contacts compared to mesophilic. The average surface density of H-bonds at thermophilic protein-rRNA interfaces (1.94 bonds per 100Å^2^ BSA) is higher than that of mesophilic (1.79 bonds per 100Å^2^ BSA). Thus, although the thermophilic and mesophilic r-proteins have almost similar structural rigidity at free cytosolic structure, thermophilic proteins undergo higher structural changes during their association with 16S rRNA, in order to generate highly complementary interfaces. This generates larger thermophilic protein-rRNA interface area and higher surface density of molecular contacts compared to mesophilic. All such attributes likely indicate the fact that thermophilic SSU is gaining extra stability compared to mesophilic in order to persist in high temperature environment.

### Essentiality of disorder-to-order transitions of thermophilic SSU-proteins

#### Understanding Disorder-to-Order and Order-to-Order transitions of r-proteins during SSU assembly

Thermophilic proteins involved in translation, transport, regulation of transcription and ribosome biogenesis differ from the rest of thermophilic proteome in the sense that they have a much higher level of disorder in hyperthermophiles than in the mesophiles [27]. In general, intrinsic disorder corresponds to generate larger interface areas within small protein and genome size [39]. The homologous r-proteins of *T. thermophilus* and *E. coli* have almost similar lengths (e.g., *T. thermophilus* and *E. coli* S13 protein has 126 and 118 amino acid chain length respectively). Instead, their rRNA interface sizes may be significantly different (e.g., in case of S3, the rRNA-interface size is 2155.44Å^2^ in *T. thermophilus* and 1808.47Å^2^ in *E. coli*). We hypothesize that longer disordered regions of thermophilic r-proteins might be correlated with their higher conformational changes and generation of highly complementary interfaces and larger BSA. Hence, the essentiality of disordered regions of thermophilic r-proteins should be reflected in their evolutionary conservation.

Disorder is generally thought to be a property of protein sequence and the disordered regions of proteins often show a disorder-to-order transition as they associate with other binding partner [40]. The thermodynamic definition of disorder in a polypeptide chain is the ‘random coil’ structural state. The random coil state is the structural ensemble spanned by a given polypeptide in which all degrees of freedom are used within the conformational space [40]. Bellay et al. [41] have shown that r-proteins merely exhibit ‘constrained disorder,’ (disorder is associated with high sequence conservation) which does not necessarily imply unstructured uncomplexed states. Therefore, disorder-to-order transition is a relative term for the r-proteins, based on their utilized degrees of freedom before and after complexation. Let us consider a residue of any r-protein, which is disordered in the free cytosolic state. Once this residue interacts with any binding partner (physical contact), its dynamics becomes dependent on the dynamics of the binding partner. In other words, interaction with a binding partner reduces the degrees of freedom of a disordered residue. Thus, according to the thermodynamic definition, the residue in the complexed state becomes more ordered compared to its uncomplexed state.

We have used the DisEMBL server [40] to predict the disordered residues of the SSU r-proteins at their uncomplexed states. Except the disordered residues, the rest are considered as the ordered residues. From this data, we have filtered out only those regions, for which the atomic coordinates are available in the crystal structures. We consider that disorder-to-order (D2O) or order-to-order (O2O) transitions occur in those amino acid residues, which become buried after the corresponding protein binds with the rRNA. A buried residue is defined as one, which looses a minimum of 1Å^2^ accessible surface area during its transition from the uncomplexed state (protein only) to complexed state (SSU). If any predicted disordered residue is found buried in the SSU structure, we have considered that it has experienced a D2O transition. On the other hand, if a predicted ordered residue is found buried in the SSU structure, we consider that it has experienced an O2O transition. In the O2O transition, the corresponding residue experiences a transition from one ordered state (free cytosolic protein) to another ordered state (protein-rRNA complex).

#### Sequence conservation at the disordered and ordered sites of thermophilic and mesophilic r-proteins

We have analyzed the sequence conservation at disordered and ordered sites of thermophilic and mesophilic r-proteins at their corresponding phylogenetic clusters (Deinococcus-Thermus and Gammaproteobacteria respectively). Although there are several methods to define the disordered region of a protein (e.g., Russell/Linding, Loops/Coil, Hot Loops [40], [42] etc.), we have used only the Loops/Coil definition of disorder (see results in Table S6, S7) since it is the closest one to the thermodynamic definition of intrinsic disorder.

The sequence conservation shows that disordered regions are generally more conserved than the ordered regions of r-proteins, which follows from the work of Bellay et al. [41]. However, they have not analyzed and discussed it in details for the individual proteins. Here, we see that the disordered regions in many of the r-proteins are significantly more conserved than the ordered regions (significance is estimated using Mann-Whitney U-test at 95% significance level). Six mesophilic (S2, S7, S11, S13, S14, and S19) and ten thermophilic (S2, S3, S7, S10, S11, S13, S14, S17, S19, and S20) SSU r-proteins (out of 21) exhibit this attribute (Table S6). The S20 is an ordered protein in *E. coli*. On the other hand, mesophilic S6 and S16 show significantly high conservation at disordered regions, compared to that at ordered regions, but their thermophilic homologs do not exhibit such difference. S4 protein shows an interesting behavior. Both the mesophilic and thermophilic S4 protein shows significantly higher evolutionary conservation at ordered regions, compared to the disordered regions (p<0.001). *T. thermophilus* S6 contains a disordered C-terminal extension (93–101), which is very lowly conserved; we excluded this region from our calculations. However, this does not alter the main observation. Similarly, extensions from some other thermophilic proteins were also excluded from our calculations due to the same reason (S8 (65–78), S17 (85–105), and S18 (1–20)). Only S17 protein (after exclusion of lowly conserved region) has a higher conservation in disorder-to-order transition than order-to-order transition; however, the difference is marginally significant. Table 2 lists the SSU r-proteins and the nature of the sequence conservation at their disordered and ordered sites.

**Table 2:** The correlation between sequence conservation at disordered and ordered sites of individual r-proteins are shown in this table.

Phylogenetic cluster	Disorder>Order (significant)	Disorder>Order (not significant)	Order>Disorder (significant)	Order>Disorder (not significant)
Gammaproteobacteria	S2, S7, S11, S13, S14, S16, S19	S5, S9, S15, S17	S4, S6	S3, S8, S10, S12, S18
Deinococcus-Thermus	S2, S3, S7, S10, S11, S13, S14, S17, S19, S20	S5, S6, S9, S12, S15, S16, S18	S4	S8

The Disorder>Order sign indicates that average conservation score at disordered region in higher than that at ordered region and so on. The ‘significant’ terminology used here describes whether the two populations (e.g. conservation scores at disordered and ordered sites) are significantly different (in U-test, p<0.05 at least) for the corresponding protein. For example, ‘Disorder>Order (not significant)’ means although average conservation score at the disordered site is higher than that at ordered site, but the two populations do not differ significantly.

#### D2O sites are more conserved than O2O sites

Thus, some mesophilic and most thermophilic r-proteins show an evolutionary tendency that disordered regions are more conserved than the ordered regions (constrained disorder). Next, we concentrated on the conservation at D2O and O2O sites of r-proteins. Both D2O and O2O transition regions are rRNA binding sites of r-proteins. However, D2O transitions would likely generate high complementary rRNA interfaces and larger interface areas; thus, causing highly stable protein-rRNA binding (essential for survival at high temperature).This structural importance of D2O sites over O2O sites should be reflected in their evolutionary conservation. We have observed this high sequence conservation at D2O sites is present (Table S7) in eight *T. thermophilus* r-proteins, whereas only four *E. coli* r-proteins exhibit this trend. This is discussed in details in the following section. Table 3 lists the SSU r-proteins and the nature of the sequence conservation at their D2O and O2O sites.

**Table 3:** The correlation between sequence conservation at D2O and O2O sites of individual r-proteins has been presented in this table.

Phylogenetic cluster	D2O>O2O (significant)	D2O>O2O (not significant)	O2O>D2O (significant)	O2O>D2O (not significant)
Gammaproteobacteria	S13, S14	S2, S3, S7, S9, S11	S4, S6	S5, S8, S10, S12, S15, S16, S17, S18
Deinococcus-Thermus	S2, S3, S6, S7, S9, S11, S14, S17	S13, S15, S16, S18, S19	S4	S5, S8, S10, S12

Terminologies are the same as Table 2.

#### High D2O conservation is observed in higher number of *T. thermophilus* r-proteins

Four mesophilic (S2, S6, S13, and S14) and seven thermophilic (S2, S3, S6, S9, S11, S14, and S17) r-proteins (out of 21) exhibit significantly high sequence conservation at their D2O transition regions than their O2O transition regions (Table S7). Therefore, higher number of *T. thermophilus* r-proteins exhibit high D2O conservation compared to those of *E. coli*. This probably entails that D2O transitions are structurally more important (for protein-rRNA interactions of 30S subunit) in thermophilic bacteria than mesophilic. The S4 protein exhibits an exception to this trend. Both thermophilic and mesophilic S4 protein shows high evolutionary conservation at the O2O regions, compared to D2O regions. S12 is a special case, which is completely disordered in its free cytosolic state (according to experimental results [43]). This might be the reason that no significant difference of evolutionary conservation is observed between its predicted disordered and ordered regions (or D2O and O2O transition regions). Thermophilic S9 protein shows no significantly high conservation in its disordered region compared to the ordered region, but the D2O transition regions are more conserved (difference is marginally significant: 0.01<p<0.05) compared to the O2O transition region. In Tables 2 and 3, we see an interesting trend that although eleven mesophilic proteins exhibit higher sequence conservation at disordered regions (compared to the ordered regions), but only two of them (18%) exhibit significantly high conservation at D2O sites (compared to O2O sites). On the other hand, seventeen thermophilic proteins exhibit high conservation at disordered regions (compared to the ordered regions), out of which eight proteins (47%) exhibit significantly high conservation at D2O sites (compared to O2O sites). Therefore, it seems, enhancement of sequence conservation at disordered and D2O sites (compared to ordered and O2O sites, respectively) is a significant attribute of thermal adaptation.

### Polarity and Disorder are conserved preferably at protein-rRNA interfaces

We have mentioned that significant reduction in the frequency of the thermolabile amino acids (histidine, glutamine, and threonine) and increment in the frequency of charged residues (arginine, lysine etc) is a common signature of thermal adaptation [18], [19]. On the other hand, intrinsically disordered proteins are also rich in charged residues and deficient in hydrophobic residues [44], [45]. Since disorder is correlated with the significantly high presence of charged residues, we assume that this might result thermophilic protein-rRNA interfaces to be more polar compared to those of mesophilic.

Except S8, S10, S11, S14, and S18, rest of the thermophilic r-proteins shows higher rRNA-interface polarity compared to their mesophilic homologs. Out of these five exceptions, three proteins (S8, S10, and S18) do not exhibit significantly high evolutionary conservation at D2O sites compared to O2O sites in either species. Thermophilic r-proteins altogether shows 64.5% rRNA-interface polarity, whereas mesophilic r-proteins exhibit 62.3% polarity. The high Arg/Lys content of rRNA buried extensions of thermophilic r-proteins [16] is likely responsible for this high polarity. The positively charged Arg/Lys preferably forms H-bonds with negatively charged rRNA backbone. This again causes low base/backbone ratio of thermophilic protein-rRNA H-bonds (0.75) compared to that of mesophilic (0.83). Now, we defined a parameter Charge Conservation Factor (CCF) as:
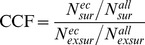
Here, in the numerator, 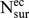
is the total number of rRNA-interface amino acid residues, which either are conserved or are replaced (in evolution) by another amino acid of equivalent charge. 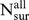
is the total number of replacements of all interface residues. In the denominator, 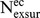
is the total number of amino acid residues (excluding interface), which either are conserved or are replaced (in evolution) by another amino acid of equivalent charge. 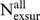
is the total number of replacements of all amino acid residues (excluding interface). CCF>1 indicates that there is an evolutionary tendency to maintain charged residues preferably at the rRNA interfaces of the proteins, than that at protein interiors. Within the bacterial domain, average CCF of the SSU r-proteins is 1.33. Proteins having large globular domains often show high CCF values (S2, S3, S4 etc.), while those having long extensions and small globular domains often show small CCF values (S12, S14). The polarity and CCF values of r-proteins are listed in Table S8.

This result clearly indicates that polar residues are preferably conserved at the rRNA-interfaces, compared to that at protein-interiors. On the other hand, we have seen that D2O transitions (disordered regions are enriched in charged residues) of r-proteins are often highly conserved. We have investigated whether presence of high polarity is correlated with longer D2O transition regions. The %polarity data of homologous thermophilic and mesophilic r-proteins are used to estimate XPOLARITY values (according to X-factor defined in “High Protein-rRNA interface complementarity is associated with larger interface areas”). If the lengths of D2O transition region of any homologous r-protein are 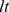
and 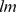
in thermophilic and mesophilic bacteria respectively, then 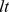
and 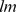
values are used to calculate the X-factor, denoted as XL-D2O. Linear correlation coefficient between XPOLARITY and XL-D2O is 0.73 (Table S9), which clearly indicates that longer D2O transition regions correspond to higher interface polarity in thermophilic r-proteins (compared to mesophilic). The essentiality of D2O transitions is likely reflected in CCF>1 values of r-proteins. In other words, CCF>1 values likely indicate that disorder is preferably conserved at protein-rRNA interfaces, compared to that at the protein interior.

## Conclusion

In this current work, we have compared a number of structural and evolutionary attributes of *E. coli* and *T. thermophilus* and have discussed their correlation with the thermal adaptation strategy of *T. thermophilus*. Within the 16S rRNA, with few exceptions, E. coli cavities are numerous and larger compared to those of thermophilic at the analogous spatial regions. In addition, we have identified many species-specific cavity clusters in both the species. Here, again we see that mesophilic cavities are numerous and larger than the thermophilic cavities. Thus, the thermophilic 16S rRNA has denser interior than that of mesophilic, which is likely a signature of thermostability.

In case of r-proteins, we have identified that thermophilic proteins generally bind with 16S rRNA ensuring high interface complementarity, which is likely correlated with large interface areas and higher surface density of molecular contacts. Homologous r-proteins of *E. coli* and *T. thermophilus* have almost similar structural rigidity at their free cytosolic state. However, thermophilic proteins have longer disordered regions and they experience higher D2O transition during SSU assembly. This D2O transition takes place in mesophilic as well, but at a reduced amount. Longer D2O transition of thermophilic r-proteins likely results their high conformational changes during SSU assembly (compared to mesophilic), which should be correlated with generation of high complementary interfaces. Generation of larger interface size and numerous molecular contacts between the constituents of thermophilic SSU ensures its higher stability compared to mesophilic. This enriched stability is essential for persisting at high temperature. The importance of D2O transition is reflected in its evolutionary conservation. This disorder, which is accompanied by the high Arg/Lys content of the r-proteins, is likely correlated with high polar thermophilic protein-rRNA interfaces (compared to mesophilic). In every r-proteins, we have identified an evolutionary tendency that polarity (hence, disorder) is preferably conserved at their rRNA interfaces, compared to that at protein interior.

Here, we should also mention that our analysis and conclusion is based on only one mesophilic and one thermophilic species. The crystal structures of other thermophilic species are not available till date. We have used a large number of available quality crystal structures of both the species to draw our conclusion. We further believe that one can get deeper insight with higher confidence once the good quality of crystal structures of other thermophilic species would be available.

## Methods

A large dataset of twenty high-resolution bacterial SSU structures (eleven from *T. thermophilus*, nine from *E. coli*) along with a eukaryotic SSU structure (*Tetrahymena thermophila*) and nine free r-protein structures (only single proteins crystallized) from different species were collected from PDB. Using this dataset, we performed the following calculations: (1) estimating the protein-rRNA and protein-protein interface size, (2) the solvent free energy of interface formation, (3) interface polarity and molecular contacts between the constituents of SSU components, (4) predicting conformational changes of proteins as they associate with SSU, (5) finding the correlation between conformational changes, interface size and solvent free energy of rRNA interactions of r-proteins, (6) estimating the nature of rRNA and r-protein interior cavities, (7) estimating the protein-rRNA interface complementarity, (8) predicting disordered and ordered regions of r-proteins and from crystal structures, predicting regions those experiences disorder-to-order and order-to-order transitions during SSU association, (9) estimating the evolutionary conservation in disordered and ordered regions. See File S2 for details.

Molecular diagrams are prepared using VMD [46]. Plots are generated using Origin Data analysis and Visualization Workspace (OriginLab Corporation).

## Supporting Information

Figure S1 The position of non-SSCC cavity clusters in mesophilic SSU, where the mesophilic 16S rRNA has denser local packing compared to that of thermophilic, are presented here. For these clusters, (CV(thermo)/CV(coli) ratio>1. Cavities are presented as red spheres, and sphere sizes are not according to cluster volume. The 16S rRNA is shown as white cartoon view, whereas proteins are shown as green cartoon view. Most of such cavities are located at the top portions of SSU, which binds with the 50S particle.
(TIF)

Figure S2 The SSU structure (A) back side view (B) 30S–50S interface side view. The A, P and E-sites are marked accordingly. The 16S rRNA is shown as sticks view, with the four domains (5′ -Domain, Central Domain, 3′-Major and 3′-minor Domains) are properly marked. The SSU r-proteins are shown as cartoon view. Homologous r-proteins having higher rRNA-interface complementarity in *T. thermophilus* are shown in blue, while those shown in green have higher interface complementarity in *E. coli*. Those homologous r-proteins of *E. coli* and *T. thermophilus* having very comparable rRNA-interface complementarity are shown in red. S10 and S12 are among this group, although both have a slight tendency of having higher interface complementarity in thermophilic.
(TIF)

Figure S3 The 3D surface plot for ΔG, RMSD and BSA is shown here. The surface is presented as 3D colormap, with projection on the BSA-RMSD plane shows the contours (each contour is for -10 Kcal/mole change of ΔG) of constant ΔG. Black dots represent the data points (not all are visible as some data points are beneath the surface). This plot is generated by Origin data analysis and graphic workspace, using the ribosomal protein-RNA association data from two bacteria (*E. coli* and *T. thermophilus*) and one eukaryotic species (*T. thermophila*).
(TIF)

Table S1 The behavior of non-SSCC cavities within the analogous spatial regions of 16S rRNA of *Escherichia coli* and *Thermus thermophilus*. We have presented the number and cluster volumes of *E. coli* and *T. thermophilus* cavities within each clusters.
(DOC)

Table S2 The cavity index and the average cavity sphericity values for *Escherichia coli* and *Thermus thermophilus* SSU proteins.
(DOC)

Table S3 The buried surface area of the ribosomal proteins for *Thermus thermophilus* and *Escherichia coli*.
(DOC)

Table S4 The constant terms (a, b and c) and the surface fitting statistics of three parameters of ribosomal proteins: Free energy of association, Buried surface area and RMSD^2^ between complexed and uncomplexed states.
(DOC)

Table S5 The free energy of association of the SSU proteins with the 16S rRNA for *Thermus thermophilus* and *Escherichia coli*.
(DOC)

Table S6 The Intrinsically Disordered/Ordered regions of *Escherichia coli* and *Thermus thermophilus* universal SSU proteins and their evolutionary conservations are presented in this table. Loops/Coil definition of disorder is used in this table. Abbreviations used: DR =  Disordered regions, ADCS =  Average Disorder Conservation Score, AOCS =  Average Order Conservation Score, p =  Mann Whitney U-test p-value (test between conservation scores of ordered and disordered residues). Significance abbreviations used: S =  significant difference (p<0.01), MS =  Marginally Significant difference (0.01<p<0.05) and N =  No difference (p>0.05) between the two populations. We assumed if disordered regions are <1% of the whole protein length, statistical calculations cannot identify significant difference. This is mentioned by the words “too small” in corresponding DR columns. Otherwise, they are left blank.
(DOC)

Table S7 The Disorder-to-Order and Order-to-Order transition regions of *Escherichia coli* and *Thermus thermophilus* universal SSU proteins and their evolutionary conservations are presented in this table. Loops/Coil definition of disorder is used in this table. Abbreviations used: D2O =  Disorder to Order transition regions, AD2OCS =  Average Disorder to Order Conservation Score, AO2OCS =  Average Order to Order Conservation Score, p =  Mann Whitney U-test p-value (test between conservation scores of ordered and disordered residues). Significance abbreviations used: S =  significant difference (p<0.01), M =  Marginal difference (0.01<p<0.05) and N =  No difference (p>0.05) between the two populations. We assumed if disordered regions are <1% of the whole protein length, statistical calculations cannot identify significant difference. This is mentioned by the words “too small” in corresponding DTO columns. Otherwise, they are left blank.
(DOC)

Table S8 The interface polarity and CCF values for the SSU r-proteins.
(DOC)

Table S9 The X_POLARITY_ and X_L-D2O_ values for r-proteins. Linear correlation coefficient calculated between these two parameters is 0.97. *E. coli* S19 and S20 proteins have no D2O transition regions, which is the reason that in these two cases we have X_L-D2O_ = 200. If we exclude S19 and S20 from our calculations, we see linear correlation coefficient between these two parameters is 0.73, which still agrees fine with our statement.
(DOC)

File S1 The dataset of 21 small ribosomal subunits and 9 ribosomal proteins used in our analysis is presented here.
(DOC)

File S2 The detailed methods and corresponding references are included here.
(DOC)

File S3 The Cavity Clustering Dendogram in NEX format.
(NEX)
