# Supplementary figures and images for: A Comparison of Structural and Evolutionary Attributes of Escherichia coli and Thermus thermophilus Small Ribosomal Subunits: Signatures of Thermal Adaptation
Source: PLoS One. 2013 Aug 5;8(8):e69898. doi: 10.1371/journal.pone.0069898 (PMC3734280; doi:10.1371/journal.pone.0069898)

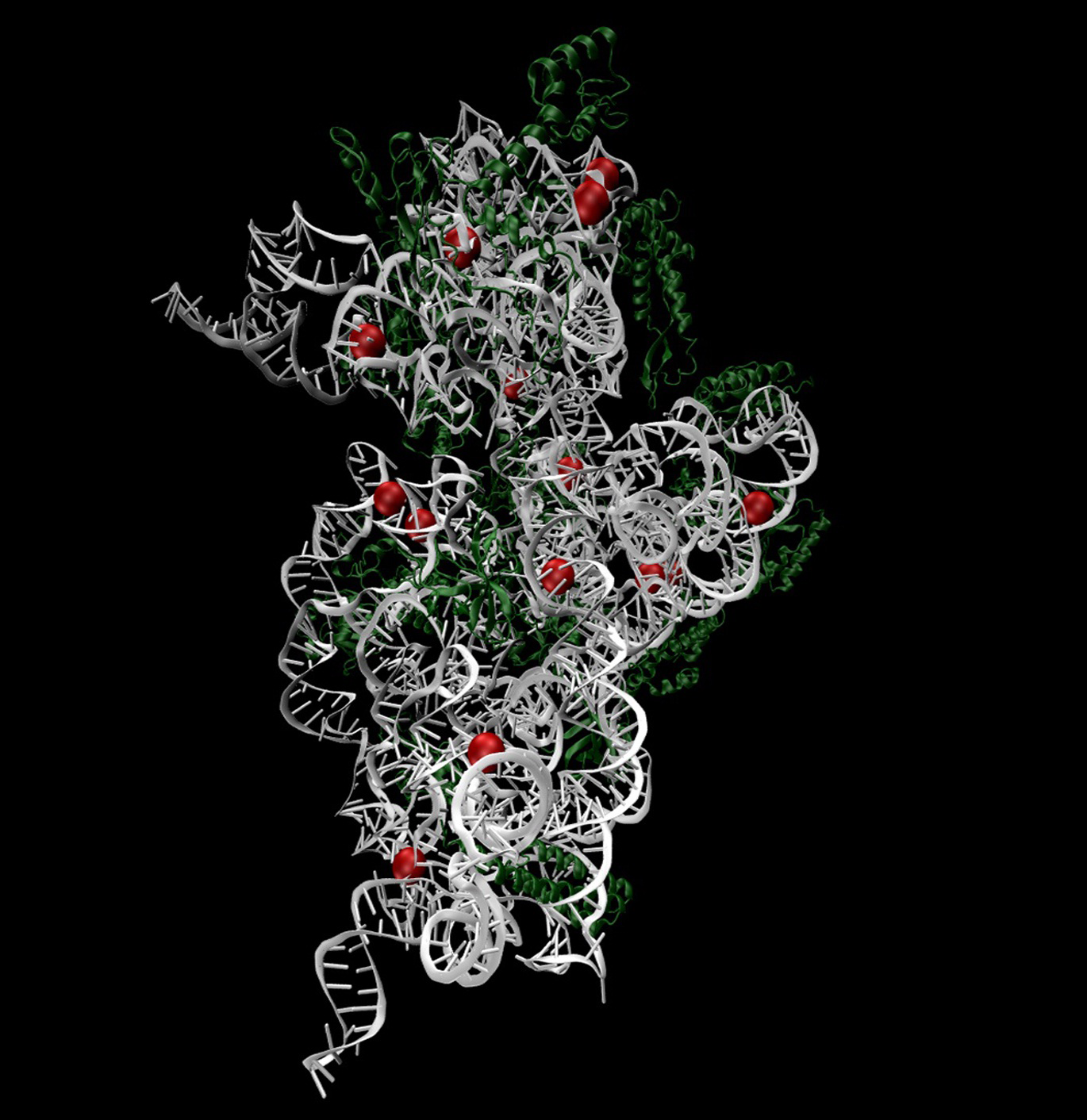

Supplement: Figure S1 — The position of non-SSCC cavity clusters in mesophilic SSU, where the mesophilic 16S rRNA has denser local packing compared to that of thermophilic, are presented here. For these clusters, (CV(thermo)/CV(coli) ratio>1. Cavities are presented as red spheres, and sphere sizes are not according to cluster volume. The 16S rRNA is shown as white cartoon view, whereas proteins are shown as green cartoon view. Most of such cavities are located at the top portions of SSU, which binds with the 50S particle. (TIF) [file pone.0069898.s001.tif]

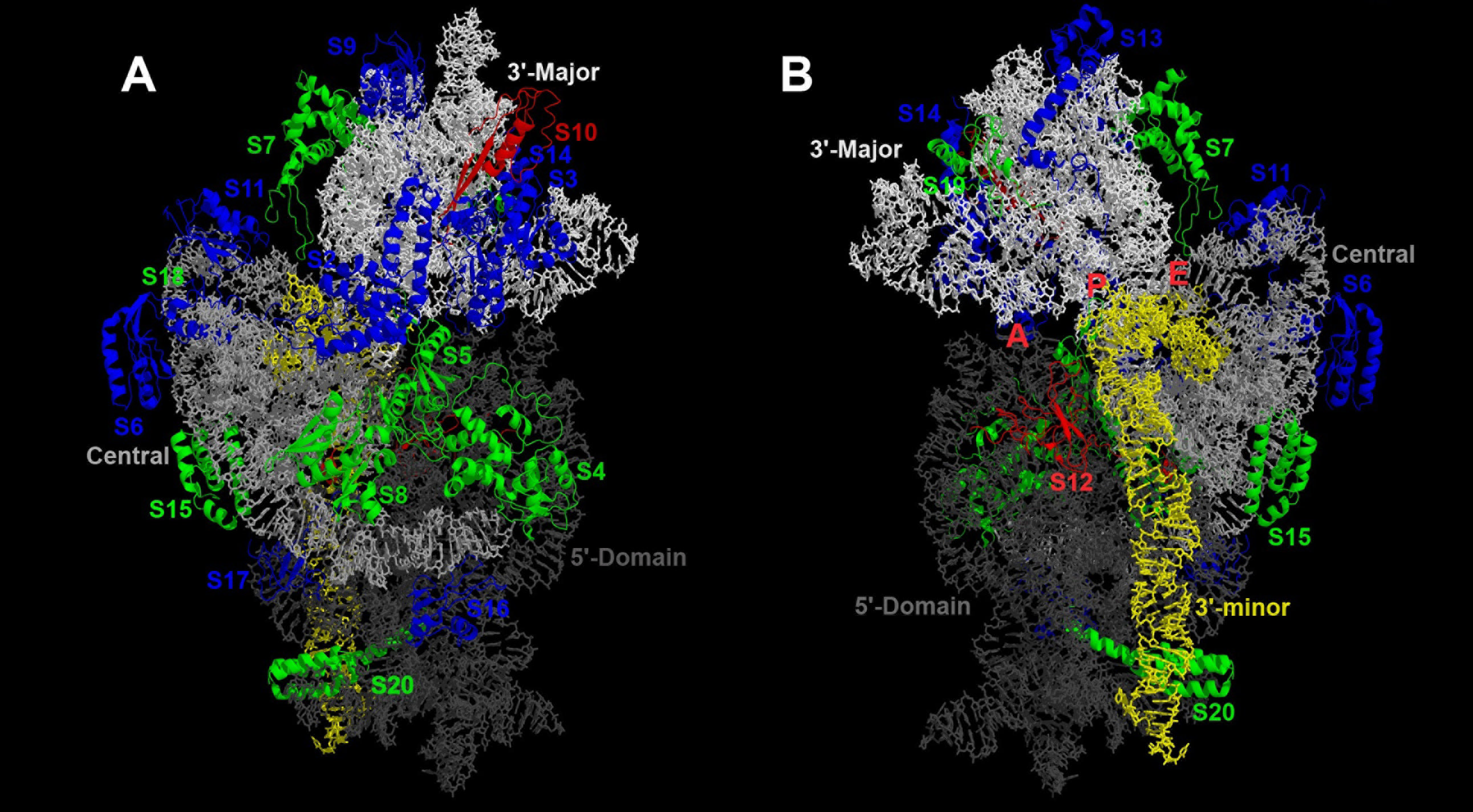

Supplement: Figure S2 — The SSU structure (A) back side view (B) 30S–50S interface side view. The A, P and E-sites are marked accordingly. The 16S rRNA is shown as sticks view, with the four domains (5′ -Domain, Central Domain, 3′-Major and 3′-minor Domains) are properly marked. The SSU r-proteins are shown as cartoon view. Homologous r-proteins having higher rRNA-interface complementarity in T. thermophilus are shown in blue, while those shown in green have higher interface complementarity in E. coli. Those homologous r-proteins of E. coli and T. thermophilus having very comparable rRNA-interface complementarity are shown in red. S10 and S12 are among this group, although both have a slight tendency of having higher interface complementarity in thermophilic. (TIF) [file pone.0069898.s002.tif]

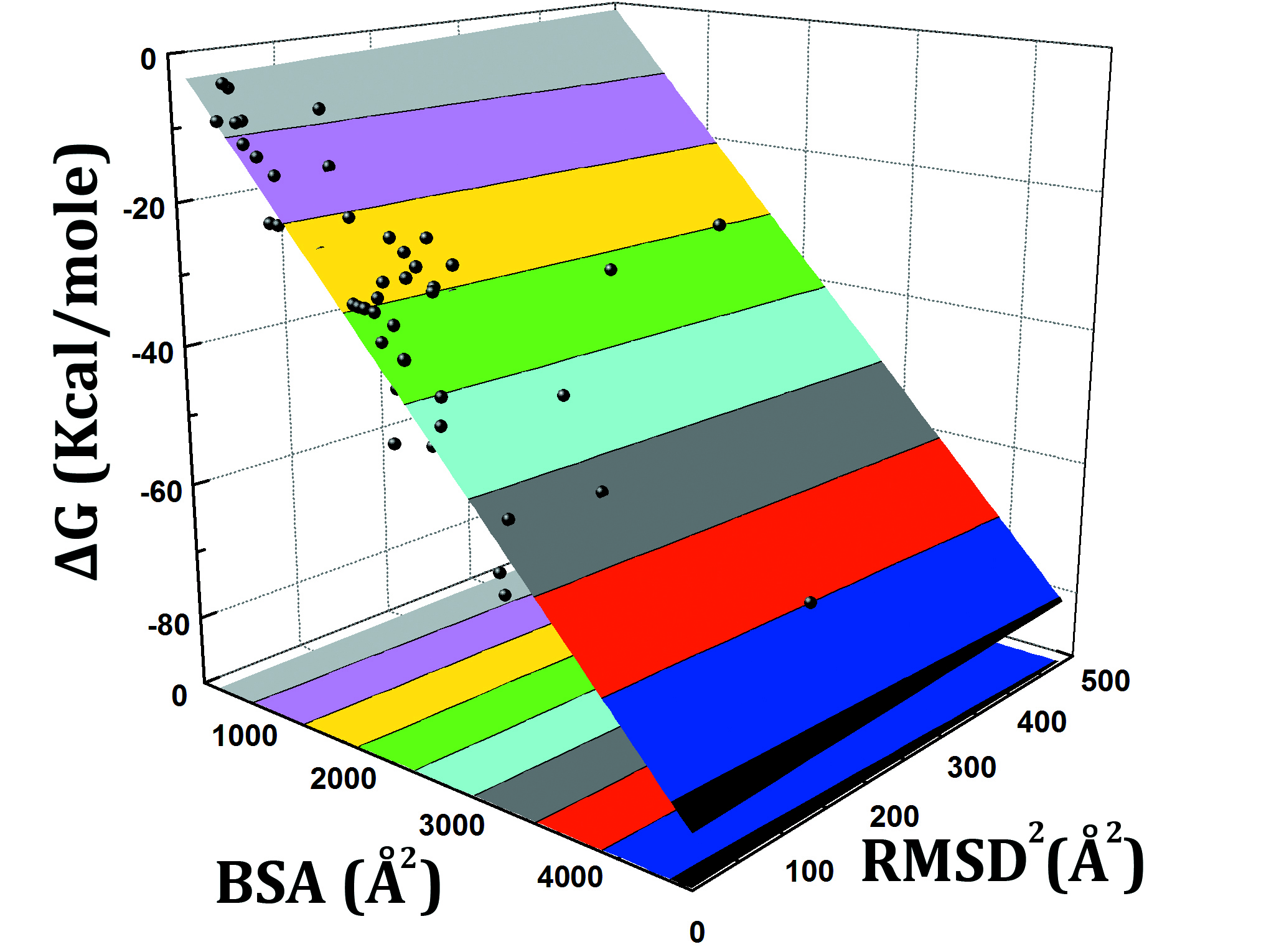

Supplement: Figure S3 — The 3D surface plot for ΔG, RMSD and BSA is shown here. The surface is presented as 3D colormap, with projection on the BSA-RMSD plane shows the contours (each contour is for -10 Kcal/mole change of ΔG) of constant ΔG. Black dots represent the data points (not all are visible as some data points are beneath the surface). This plot is generated by Origin data analysis and graphic workspace, using the ribosomal protein-RNA association data from two bacteria (E. coli and T. thermophilus) and one eukaryotic species (T. thermophila). (TIF) [file pone.0069898.s003.tif]
