# Supplementary material for: A Comparison of Structural and Evolutionary Attributes of Escherichia coli and Thermus thermophilus Small Ribosomal Subunits: Signatures of Thermal Adaptation
Source: PLoS One. 2013 Aug 5;8(8):e69898. doi: 10.1371/journal.pone.0069898 (PMC3734280; doi:10.1371/journal.pone.0069898)
Supplement: Table S1 — The behavior of non-SSCC cavities within the analogous spatial regions of 16S rRNA of Escherichia coli and Thermus thermophilus. We have presented the number and cluster volumes of E. coli and T. thermophilus cavities within each clusters. (DOC) [file pone.0069898.s004.doc]

| Cluster number | Number of members | | Cluster Volume (Å^3^) | | Ratio of Cluster Volumes |
| --- | --- | --- | --- | --- | --- |
|  | *Escherichia coli* | *Thermus thermophilus* | *Escherichia coli* | *Thermus thermophilus* |  |
| 1 | 2 | 1 | 25.86 | 17.83 | 0.69 |
| 2 | 4 | 1 | 169.56 | 44.03 | 0.26 |
| 3 | 3 | 1 | 428.63 | 67.90 | 0.16 |
| 4 | 4 | 1 | 106.79 | 15.71 | 0.15 |
| 5 | 2 | 4 | 253.48 | 124.61 | 0.49 |
| 6 | 1 | 2 | 16.32 | 35.32 | 2.16 |
| 7 | 2 | 1 | 78.97 | 46.74 | 0.59 |
| 8 | 1 | 2 | 11.60 | 24.99 | 2.15 |
| 9 | 1 | 2 | 27.15 | 14.04 | 0.52 |
| 10 | 2 | 3 | 147.76 | 129.66 | 0.88 |
| 11 | 3 | 2 | 361.83 | 40.11 | 0.11 |
| 12 | 2 | 1 | 46.48 | 26.43 | 0.57 |
| 13 | 1 | 1 | 44.17 | 16.49 | 0.37 |
| 14 | 2 | 1 | 69.69 | 11.67 | 0.17 |
| 15 | 2 | 2 | 117.28 | 28.54 | 0.24 |
| 16 | 2 | 1 | 32.64 | 28.53 | 0.87 |
| 17 | 2 | 2 | 29.73 | 57.15 | 1.92 |
| 18 | 1 | 1 | 28.72 | 12.91 | 0.45 |
| 19 | 2 | 1 | 321.75 | 289.49 | 0.90 |
| 20 | 2 | 1 | 124.37 | 57.69 | 0.46 |
| 21 | 3 | 1 | 113.31 | 166.27 | 1.47 |
| 22 | 2 | 2 | 24.96 | 29.20 | 1.17 |
| 23 | 2 | 3 | 27.46 | 101.83 | 3.71 |
| 24 | 2 | 2 | 106.09 | 108.20 | 1.02 |
| 25 | 2 | 2 | 117.27 | 112.16 | 0.96 |
| 26 | 1 | 1 | 18.11 | 11.97 | 0.66 |
| 27 | 2 | 4 | 57.55 | 86.46 | 1.50 |
| 28 | 4 | 1 | 128.10 | 33.37 | 0.26 |
| 29 | 1 | 1 | 24.13 | 32.71 | 1.36 |
| 30 | 2 | 1 | 77.32 | 15.23 | 0.20 |
| 31 | 1 | 1 | 39.79 | 29.13 | 0.73 |
| 32 | 1 | 1 | 19.88 | 33.97 | 1.71 |
| 33 | 2 | 1 | 26.13 | 16.65 | 0.64 |
| 34 | 2 | 1 | 23.51 | 19.16 | 0.81 |
| 35 | 1 | 1 | 45.05 | 26.70 | 0.59 |
| 36 | 2 | 1 | 39.04 | 78.16 | 2.00 |
| 37 | 1 | 1 | 13.51 | 44.74 | 3.31 |
| 38 | 1 | 3 | 14.54 | 136.21 | 9.37 |
| 39 | 2 | 2 | 44.09 | 36.32 | 0.82 |
| 40 | 3 | 2 | 66.25 | 44.40 | 0.67 |
| 41 | 2 | 1 | 27.62 | 22.09 | 0.80 |
| 42 | 1 | 2 | 63.69 | 61.03 | 0.96 |
| 43 | 3 | 1 | 50.85 | 11.97 | 0.24 |
| 44 | 1 | 2 | 83.67 | 124.19 | 1.48 |
| 45 | 3 | 1 | 39.73 | 12.18 | 0.31 |
