# Supplementary material for: A Comparison of Structural and Evolutionary Attributes of Escherichia coli and Thermus thermophilus Small Ribosomal Subunits: Signatures of Thermal Adaptation
Source: PLoS One. 2013 Aug 5;8(8):e69898. doi: 10.1371/journal.pone.0069898 (PMC3734280; doi:10.1371/journal.pone.0069898)
Supplement: Table S2 — The cavity index and the average cavity sphericity values for Escherichia coli and Thermus thermophilus SSU proteins. (DOC) [file pone.0069898.s005.doc]

| Ribosomal proteins | *Escherichia coli* proteins | | *Thermus thermophilus* proteins | |
| --- | --- | --- | --- | --- |
|  | Cavity index | Average Cavity Spehericity | Cavity index | Average Cavity Spehericity |
| S2 | 0.16 | 0.929 | 0.11 | 0.957 |
| S3 | 0.18 | 1.262 | 0.09 | 0.360 |
| S4 | 0.44 | 0.937 | 0.62 | 0.919 |
| S5 | 0.07 | 0.944 | 0.14 | 0.931 |
| S6 | 0.17 | 0.980 | 0.00 | - |
| S7 | 0.05 | 0.937 | 0.11 | 0.939 |
| S8 | 0.17 | 1.033 | 0.20 | 0.970 |
| S9 | 0.18 | 1.091 | 0.09 | 0.927 |
| S10 | 0.67 | 0.854 | 0.66 | 0.899 |
| S11 | 0.44 | 0.930 | 0.31 | 0.936 |
| S12 | 0.16 | 0.856 | 0.14 | 0.996 |
| S13 | 0.40 | 0.956 | 0.23 | 1.996 |
| S14 | 0.16 | 0.919 | 0.05 | 0.988 |
| S15 | 0.11 | 1.133 | 0.23 | 0.951 |
| S16 | 0.34 | 1.175 | 0.05 | 0.957 |
| S17 | 0.69 | 1.023 | 0.21 | 0.954 |
| S18 | 0.04 | 0.933 | 0.02 | 0.992 |
| S19 | 0.12 | 0.897 | 0.50 | 1.147 |
| S20 | 0.06 | 1.094 | 0.15 | 1.040 |
| S21 | 0.03 | 0.961 | - | - |
| THX | - | - | 0.03 | 0.976 |
| S3-S10-S14 | - | - | 0.13 | 0.939 |
| S9-S13-S19-THX | - | - | 0.25 | 1.004 |
| S4-S5 | 0.14 | 0.943 | - | - |
| S6-S11-S18 | 0.41 | 0.933 | - | - |
| S8-S12 | 0.08 | 0.942 | - | - |
| S9-S10-S13-S14-S19 | 0.19 | 0.958 | - | - |
