# Supplementary material for: A Comparison of Structural and Evolutionary Attributes of Escherichia coli and Thermus thermophilus Small Ribosomal Subunits: Signatures of Thermal Adaptation
Source: PLoS One. 2013 Aug 5;8(8):e69898. doi: 10.1371/journal.pone.0069898 (PMC3734280; doi:10.1371/journal.pone.0069898)
Supplement: Table S3 — The buried surface area of the ribosomal proteins for Thermus thermophilus and Escherichia coli. (DOC) [file pone.0069898.s006.doc]

| Ribosomal proteins | Average BSA of *T. thermophilus* proteins (Å^2^) | Standard Deviation | BSA of *E. coli* proteins (Å^2^) | Standard deviation |
| --- | --- | --- | --- | --- |
| S2 | 998.71 | 74.43 | 937.12 | 88.90 |
| S3 | 1691.35 | 56.04 | 1622.40 | 86.32 |
| S4 | 2965.99 | 243.30 | 3006.92 | 93.88 |
| S5 | 1959.26 | 53.15 | 1769.82 | 54.93 |
| S6 | 647.18 | 189.11 | 432.66 | 30.73 |
| S7 | 1859.82 | 76.37 | 1790.46 | 84.70 |
| S8 | 1874.48 | 77.83 | 1742.46 | 24.97 |
| S9 | 2943.62 | 173.86 | 2836.02 | 43.67 |
| S10 | 1801.79 | 40.92 | 1742.60 | 27.61 |
| S11 | 1825.82 | 31.41 | 2000.72 | 6.52 |
| S12 | 3106.87 | 118.09 | 2751.24 | 151.00 |
| S13 | 2155.44 | 128.88 | 1808.47 | 49.70 |
| S14 | 1931.28 | 99.15 | 2072.64 | 101.79 |
| S15 | 1776.25 | 36.98 | 1628.04 | 36.62 |
| S16 | 2505.85 | 69.35 | 2099.58 | 53.48 |
| S17 | 2669.90 | 72.11 | 1603.21 | 37.27 |
| S18 | 864.57 | 14.37 | 935.93 | 40.09 |
| S19 | 1549.82 | 162.29 | 1550.89 | 91.95 |
| S20 | 2541.10 | 88.14 | 2139.38 | 47.71 |
| S21 | - | - | 514.84 | 36.25 |
| THX | 1287.49 | 34.63 | - | - |
