# Supplementary material for: A Comparison of Structural and Evolutionary Attributes of Escherichia coli and Thermus thermophilus Small Ribosomal Subunits: Signatures of Thermal Adaptation
Source: PLoS One. 2013 Aug 5;8(8):e69898. doi: 10.1371/journal.pone.0069898 (PMC3734280; doi:10.1371/journal.pone.0069898)
Supplement: Table S4 — The constant terms (a, b and c) and the surface fitting statistics of three parameters of ribosomal proteins: Free energy of association, Buried surface area and RMSD2 between complexed and uncomplexed states. (DOC) [file pone.0069898.s007.doc]

| Constant terms | *Thermus thermophilus* | *Escherichia coli* | *Tetrahymena thermophila* | Three species together |
| --- | --- | --- | --- | --- |
| a | 2.039 | 3.394 | -0.190 | -0.311 |
| b | -0.001 | -0.012 | -0.018 | -0.018 |
| c | -0.018 | -0.020 | 0.006 | 0.005 |
| R-value of surface fitting | 0.95 | 0.94 | 0.94 | 0.94 |
| R-square value of surface fitting | 0.89 | 0.89 | 0.88 | 0.87 |
