# Supplementary material for: A Comparison of Structural and Evolutionary Attributes of Escherichia coli and Thermus thermophilus Small Ribosomal Subunits: Signatures of Thermal Adaptation
Source: PLoS One. 2013 Aug 5;8(8):e69898. doi: 10.1371/journal.pone.0069898 (PMC3734280; doi:10.1371/journal.pone.0069898)
Supplement: Table S5 — The free energy of association of the SSU proteins with the 16S rRNA for Thermus thermophilus and Escherichia coli. (DOC) [file pone.0069898.s008.doc]

| Ribosomal proteins | Solvent free energy of  association (ΔG) with 16S rRNA  (Kcal/mole) for *T. thermophilus* proteins | Standard Deviation | Solvent free energy of  association (ΔG) with 16S rRNA  (Kcal/mole) for *E. coli* proteins (Å^2^) | Standard deviation |
| --- | --- | --- | --- | --- |
| S2 | -17.58 | 4.17 | -19.28 | 2.68 |
| S3 | -28.49 | 1.79 | -32.68 | 0.85 |
| S4 | -52.60 | 2.36 | -61.79 | 3.75 |
| S5 | -33.96 | 1.56 | -30.32 | 0.97 |
| S6 | -8.18 | 0.30 | -8.10 | 0.62 |
| S7 | -25.05 | 3.47 | -26.26 | 6.02 |
| S8 | -28.46 | 0.75 | -28.06 | 1.93 |
| S9 | -54.36 | 2.20 | -51.54 | 4.52 |
| S10 | -28.37 | 3.05 | -26.65 | 2.14 |
| S11 | -32.58 | 0.84 | -34.22 | 1.45 |
| S12 | -58.13 | 1.40 | -50.99 | 1.85 |
| S13 | -42.98 | 4.91 | -42.38 | 0.75 |
| S14 | -31.68 | 3.57 | -41.24 | 2.61 |
| S15 | -37.01 | 1.67 | -29.98 | 2.27 |
| S16 | -45.88 | 4.38 | -37.11 | 2.35 |
| S17 | -42.38 | 2.03 | -33.09 | 1.36 |
| S18 | -20.17 | 0.63 | -19.02 | 1.46 |
| S19 | -19.01 | 3.26 | -19.10 | 3.58 |
| S20 | -51.56 | 1.52 | -44.66 | 1.64 |
| S21 | - | - | -9.80 | 2.06 |
| THX | -27.09 | 3.17 | - | - |
