# Supplementary material for: A Comparison of Structural and Evolutionary Attributes of Escherichia coli and Thermus thermophilus Small Ribosomal Subunits: Signatures of Thermal Adaptation
Source: PLoS One. 2013 Aug 5;8(8):e69898. doi: 10.1371/journal.pone.0069898 (PMC3734280; doi:10.1371/journal.pone.0069898)
Supplement: Table S6 — The Intrinsically Disordered/Ordered regions of Escherichia coli and Thermus thermophilus universal SSU proteins and their evolutionary conservations are presented in this table. Loops/Coil definition of disorder is used in this table. Abbreviations used: DR = Disordered regions, ADCS = Average Disorder Conservation Score, AOCS = Average Order Conservation Score, p = Mann Whitney U-test p-value (test between conservation scores of ordered and disordered residues). Significance abbreviations used: S = significant difference (p<0.01), MS = Marginally Significant difference (0.01<p<0.05) and N = No difference (p>0.05) between the two populations. We assumed if disordered regions are <1% of the whole protein length, statistical calculations cannot identify significant difference. This is mentioned by the words “too small” in corresponding DR columns. Otherwise, they are left blank. (DOC) [file pone.0069898.s009.doc]

| Universal SSU Proteins | *Escherichia coli* Proteins | | | | | *Thermus thermophilus* proteins | | | | |
| --- | --- | --- | --- | --- | --- | --- | --- | --- | --- | --- |
|  | DR | ADCS | AOCS | p | Difference significant? | DR | ADCS | AOCS | p | Difference significant? |
| S2 |  | 0.854 | 0.736 | 0.00016 | S |  | 0.759 | 0.691 | 0.0098 | S |
| S3 |  | 0.728 | 0.795 | 0.4622 | N |  | 0.836 | 0.691 | 4.56E-06 | S |
| S4 |  | 0.721 | 0.845 | 2.60E-07 | S |  | 0.560 | 0.714 | 3.92E-06 | S |
| S5 |  | 0.770 | 0.734 | 0.2968 | N |  | 0.685 | 0.639 | 0.2403 | N |
| S6 |  | 0.485 | 0.775 | 3.43E-07 | S |  | 0.624 | 0.582 | 0.4269 | N |
| S7 |  | 0.839 | 0.773 | 0.026 | MS |  | 0.791 | 0.714 | 0.0272 | MS |
| S8 |  | 0.743 | 0.797 | 0.3241 | N |  | 0.657 | 0.707 | 0.2508 | N |
| S9 |  | 0.796 | 0.788 | 0.4917 | N |  | 0.662 | 0.629 | 0.118 | N |
| S10 |  | 0.825 | 0.842 | 0.9251 | N |  | 0.681 | 0.571 | 0.0351 | MS |
| S11 |  | 0.866 | 0.758 | 0.0055 | S |  | 0.801 | 0.714 | 0.0276 | MS |
| S12 |  | 0.901 | 0.913 | 0.4567 | N |  | 0.864 | 0.856 | 0.6939 | N |
| S13 |  | 0.845 | 0.765 | 0.0392 | MS |  | 0.792 | 0.702 | 0.0146 | MS |
| S14 |  | 0.813 | 0.697 | 0.0011 | S |  | 0.707 | 0.585 | 0.033 | MS |
| S15 |  | 0.785 | 0.753 | 0.4021 | N |  | 0.751 | 0.683 | 0.2546 | N |
| S16 |  | 0.808 | 0.723 | 0.027 | MS |  | 0.660 | 0.614 | 0.305 | N |
| S17 |  | 0.736 | 0.694 | 0.4021 | N |  | 0.690 | 0.557 | 0.022 | MS |
| S18 |  | 0.708 | 0.799 | 0.1046 | N |  | 0.682 | 0.623 | 0.3642 | N |
| S19 |  | 0.852 | 0.753 | 0.0048 | S |  | 0.844 | 0.723 | 0.0035 | S |
| S20 | none | - | - | - | - |  | 0.556 | 0.469 | 0.0074 | S |
