# Supplementary material for: A Comparison of Structural and Evolutionary Attributes of Escherichia coli and Thermus thermophilus Small Ribosomal Subunits: Signatures of Thermal Adaptation
Source: PLoS One. 2013 Aug 5;8(8):e69898. doi: 10.1371/journal.pone.0069898 (PMC3734280; doi:10.1371/journal.pone.0069898)
Supplement: Table S7 — The Disorder-to-Order and Order-to-Order transition regions of Escherichia coli and Thermus thermophilus universal SSU proteins and their evolutionary conservations are presented in this table. Loops/Coil definition of disorder is used in this table. Abbreviations used: D2O = Disorder to Order transition regions, AD2OCS = Average Disorder to Order Conservation Score, AO2OCS = Average Order to Order Conservation Score, p = Mann Whitney U-test p-value (test between conservation scores of ordered and disordered residues). Significance abbreviations used: S = significant difference (p<0.01), M = Marginal difference (0.01<p<0.05) and N = No difference (p>0.05) between the two populations. We assumed if disordered regions are <1% of the whole protein length, statistical calculations cannot identify significant difference. This is mentioned by the words “too small” in corresponding DTO columns. Otherwise, they are left blank. (DOC) [file pone.0069898.s010.doc]

| Universal SSU Proteins | *Escherichia coli* Proteins | | | | | *Thermus thermophilus* proteins | | | | |
| --- | --- | --- | --- | --- | --- | --- | --- | --- | --- | --- |
|  | D2O | AD2OCS | AO2OCS | p | Difference significant? | D2O | AD2OCS | AO2OCS | p | Difference significant? |
| S2 |  | 0.857 | 0.914 | 0.123 | N |  | 0.884 | 0.726 | 0.0047 | S |
| S3 |  | 0.842 | 0.800 | 0.5043 | N |  | 0.866 | 0.730 | 0.006 | S |
| S4 |  | 0.769 | 0.846 | 0.0088 | S |  | 0.585 | 0.745 | 0.0045 | S |
| S5 |  | 0.804 | 0.844 | 0.9123 | N |  | 0.679 | 0.689 | 0.8153 | N |
| S6 |  | 0.556 | 0.844 | 0.0003 | S |  | 0.770 | 0.606 | 0.3053 | MS |
| S7 |  | 0.842 | 0.826 | 0.8525 | N |  | 0.803 | 0.742 | 0.2715 | N |
| S8 |  | 0.769 | 0.832 | 0.3296 | N |  | 0.720 | 0.740 | 0.7145 | N |
| S9 |  | 0.840 | 0.816 | 0.6643 | N |  | 0.753 | 0.649 | 0.0231 | MS |
| S10 |  | 0.823 | 0.881 | 0.493 | N |  | 0.676 | 0.677 | 0.8624 | N |
| S11 |  | 0.908 | 0.849 | 0.2298 | N |  | 0.861 | 0.721 | 0.0086 | S |
| S12 |  | 0.887 | 0.907 | 0.9401 | N |  | 0.873 | 0.874 | 0.9977 | N |
| S13 |  | 0.939 | 0.834 | 0.045 | MS |  | 0.841 | 0.779 | 0.1269 | N |
| S14 |  | 0.828 | 0.732 | 0.0156 | MS |  | 0.714 | 0.585 | 0.0424 | MS |
| S15 |  | 0.807 | 0.753 | 0.3461 | N |  | 0.743 | 0.705 | 0.5256 | N |
| S16 |  | 0.829 | 0.744 | 0.1346 | N |  | 0.699 | 0.599 | 0.1004 | N |
| S17 |  | 0.827 | 0.718 | 0.1197 | N |  | 0.695 | 0.517 | 0.0347 | MS |
| S18 |  | 0.792 | 0.777 | 0.9794 | N |  | 0.672 | 0.572 | 0.2533 | N |
| S19 |  | - | none | - | - |  | 0.873 | 0.851 | 0.6351 | N |
| S20 | none | - | - | - | - |  | - | none | - | - |
