# Supplementary material for: A Comparison of Structural and Evolutionary Attributes of Escherichia coli and Thermus thermophilus Small Ribosomal Subunits: Signatures of Thermal Adaptation
Source: PLoS One. 2013 Aug 5;8(8):e69898. doi: 10.1371/journal.pone.0069898 (PMC3734280; doi:10.1371/journal.pone.0069898)
Supplement: Table S8 — The interface polarity and CCF values for the SSU r-proteins. (DOC) [file pone.0069898.s011.doc]

| Ribosomal Proteins | Interface Polarity | | CCF |
| --- | --- | --- | --- |
|  | *T. thermophilus* | *E. coli* |
| S2 | 65.12 | 62.04 | S2 |
| S3 | 61.53 | 60.31 | S3 |
| S4 | 68.30 | 62.59 | S4 |
| S5 | 63.32 | 60.01 | S5 |
| S6 | 62.70 | 59.95 | S6 |
| S7 | 68.78 | 57.30 | S7 |
| S8 | 54.88 | 59.09 | S8 |
| S9 | 66.45 | 59.77 | S9 |
| S10 | 59.15 | 59.07 | S10 |
| S11 | 64.37 | 62.27 | S11 |
| S12 | 68.39 | 70.04 | S12 |
| S13 | 65.23 | 65.19 | S13 |
| S14 | 67.38 | 64.30 | S14 |
| S15 | 61.58 | 64.17 | S15 |
| S16 | 62.59 | 62.04 | S16 |
| S17 | 62.92 | 61.37 | S17 |
| S18 | 63.20 | 61.90 | S18 |
| S19 | 59.72 | 63.99 | S19 |
| S20 | 68.99 | 68.69 | S20 |
| THX | - | 66.98 | THX |
| S21 | 67.97 | - | S21 |
