# Supplementary material for: A Comparison of Structural and Evolutionary Attributes of Escherichia coli and Thermus thermophilus Small Ribosomal Subunits: Signatures of Thermal Adaptation
Source: PLoS One. 2013 Aug 5;8(8):e69898. doi: 10.1371/journal.pone.0069898 (PMC3734280; doi:10.1371/journal.pone.0069898)
Supplement: Table S9 — The XPOLARITY and XL-D2O values for r-proteins. Linear correlation coefficient calculated between these two parameters is 0.97. E. coli S19 and S20 proteins have no D2O transition regions, which is the reason that in these two cases we have XL-D2O = 200. If we exclude S19 and S20 from our calculations, we see linear correlation coefficient between these two parameters is 0.73, which still agrees fine with our statement. (DOC) [file pone.0069898.s012.doc]

| Ribosomal Proteins | X_POLARITY_ | X_L-D2O_ |
| --- | --- | --- |
| S2 | 8.46045 | 57.14 |
| S3 | -0.21694 | -33.85 |
| S4 | 2.98554 | 43.68 |
| S5 | 7.05805 | 4.76 |
| S6 | 13.77646 | -43.48 |
| S7 | 10.80087 | -35.29 |
| S8 | -5.29516 | 60.00 |
| S9 | 4.59915 | 8.22 |
| S10 | -2.06884 | 29.33 |
| S11 | -10.02206 | 7.41 |
| S12 | 8.48485 | -13.33 |
| S13 | 16.94915 | 43.75 |
| S14 | -21.06729 | -28.57 |
| S15 | 2.49334 | 5.41 |
| S16 | 17.40706 | 20.00 |
| S17 | 49.88783 | -14.29 |
| S18 | -19.62304 | 13.33 |
| S19 | 0.18283 | 200.00 |
| S20 | 13.29 | 200.00 |
