# Supplementary material for: A Comparison of Structural and Evolutionary Attributes of Escherichia coli and Thermus thermophilus Small Ribosomal Subunits: Signatures of Thermal Adaptation
Source: PLoS One. 2013 Aug 5;8(8):e69898. doi: 10.1371/journal.pone.0069898 (PMC3734280; doi:10.1371/journal.pone.0069898)
Supplement: File S1 — The dataset of 21 small ribosomal subunits and 9 ribosomal proteins used in our analysis is presented here. (DOC) [file pone.0069898.s013.doc]

| **A comparison of structural and evolutionary attributes of *Escherichia coli* and *Thermus thermophilus* small ribosomal subunits: signatures of thermal adaptation**  Saurav Mallik, and Sudip Kundu^*^  Department of Biophysics, Molecular Biology and Bioinformatics, University of Calcutta  Address: 92, APC Road, Kolkata; India; Zip: 700009; Email: Saurav Mallik: saurav.bmb@gmail.com; Sudip Kundu: skbmbg@caluniv.ac.in |
| --- |

**SUPPORTING INFORMATION-1**

**DATASET**

A large dataset of twenty high-resolution bacterial SSU structures (eleven from *T. thermophilus*, nine from *E. coli*) along with a eukaryotic SSU structure (*Tetrahymena thermophila*) and nine free r-protein structures from different species were collected from PDB (Bernstein et al. 1977). The list of PDB files along with their resolution and proper citation are presented in supplementary materials Table S1. We neglected all the SSU structures containing modified residues, full length tRNA, mRNA, elongation factors and release factors. Structures with ≥4.0 Å resolution are also disregarded. However, two SSU structures (1XNQ and 1IBK) contain small fragments of mRNA (3-4 nucleotides only). Unique structures containing only ribosomal RNA and ribosomal proteins (there may be antibiotics and other small molecules bound) were selected for our analysis. Yeast ribosomal complexes are available in PDB, but detectable protein-rRNA interfaces were not identified in them. Therefore, we used the only available *Tetrahymena thermophila* ribosomal structure, in spite of presence of modified residues in it.

**The dataset of 21 small ribosomal subunits and 9 ribosomal proteins used in our analysis is presented here. We have mentioned their resolutions and proper citation in the same table.**

| **Structure** | **Organism** | **PDB-ID** | **Resolution (Å)** | **Citation** |
| --- | --- | --- | --- | --- |
|  | *Thermus thermophilus* | 1FJG | 3.00 | Carter, A.P.,  Clemons Jr., W.M.,  Brodersen, D.E.,  Morgan-Warren, R.J.,  Wimberly, B.T.,  Ramakrishnan, V. (2000) Nature **407:**340-348 |
|  |  | 1N36 | 3.65 | Ogle, J.M.,  Murphy IV, F.V.,  Tarry, M.J.,  Ramakrishnan, V. (2002) , Cell(Cambridge,Mass.) **111:**721-732 |
| **30S Ribosomal Subunit** |  | 1XNQ | 3.05 | Murphy, F.V.,  Ramakrishnan, V. (2004) Nat.Struct.Mol.Biol. **11:**1251-1252 |
|  |  | 2E5L | 3.30 | Kaminishi, T.,  Wilson, D.N.,  Takemoto, C.,  Harms, J.M.,  Kawazoe, M.,  Schluenzen, F.,  Hanawa-Suetsugu, K.,  Shirouzu, M.,  Fucini, P.,  Yokoyama, S. (2007) Structure **15:**289-297 |
|  |  | 2F4V | 3.80 | Murray, J.B.,  Meroueh, S.O.,  Russell, R.J.,  Lentzen, G.,  Haddad, J.,  Mobashery, S. (2006) Chem.Biol. **13:**129-138 |
|  |  | 1IBK | 3.31 | Ogle, J.M.,  Brodersen, D.E.,  Clemons Jr., W.M.,  Tarry, M.J.,  Carter, A.P.,  Ramakrishnan, V. (2001) Science **292:**897-902 |
|  |  | 2ZM6 | 3.30 | Kaminishi, T., Wang, H., Kawazoe, M., Ishii, R., Hanawa-Suetsugu, K., Nomura, M., Takemoto, C., Shirouzu, M., Paola, F., Yokoyama, S. (to be published) |
|  |  | 3OGE | 3.00 | Bulkley, D., Innis, C.A., Blaha, G., Steitz, T.A. (2010). Revisiting the structures of several antibiotics bound to the bacterial ribosome. Proc.Natl.Acad.Sci.USA 107: 17158-17163 |
|  |  | 3UXT | 3.20 | Bulkley, D.,  Johnson, F.,  Steitz, T.A., (2012) J.Mol.Biol. 416: 571-578 |
|  |  | 3OHC | 3.00 | Bulkley, D.,  Innis, C.A.,  Blaha, G.,  Steitz, T.A., (2010) Proc.Natl.Acad.Sci.USA 107: 17158-17163 |
|  |  | 2HHH | 3.35 | Schluenzen, F.,  Takemoto, C.,  Wilson, D.N.,  Kaminishi, T.,  Harms, J.M.,  Hanawa-Suetsugu, K.,  Szaflarski, W.,  Kawazoe, M.,  Shirouzo, M.,  Nierhaus, K.H.,  Yokoyama, S.,  Fucini, P., (2006) Nat.Struct.Mol.Biol. 13: 871-878 |
|  | *Escherichia coli* | 2AVY | 3.46 | Schuwirth, B.S.,  Borovinskaya, M.A.,  Hau, C.W.,  Zhang, W.,  Vila-Sanjurjo, A.,  Holton, J.M.,  Cate, J.H. (2005) Science **310:**827-834 |
|  |  | 3OAQ | 3.25 | Dunkle, J.A.,  Xiong, L.,  Mankin, A.S.,  Cate, J.H. (2010) Proc.Natl.Acad.Sci.USA **107:**17152-17157 |
|  |  | 2QAN | 3.21 | Borovinskaya, M.A.,  Pai, R.D.,  Zhang, W.,  Schuwirth, B.S.,  Holton, J.M.,  Hirokawa, G.,  Kaji, H.,  Kaji, A.,  Cate, J.H. (2007) Nat.Struct.Mol.Biol. **14:**727-732 |
|  |  | 3DF1 | 3.50 | Borovinskaya, M.A.,  Shoji, S.,  Fredrick, K.,  Cate, J.H.D. (2008) Rna **14:**1590-1599 |
|  |  | 2VHO | 3.00 | Bingel-Erlenmeyer, R.,  Kohler, R.,  Kramer, G.,  Sandikci, A.,  Antolic, S.,  Maier, T.,  Schaffitzel, C.,  Wiedmann, B.,  Bukau, B.,  Ban, N. (2008) Nature **452:**108 |
|  |  | 2QP0 | 3.50 | Borovinskaya, M.A., Shoji, S., Holton, J.M., Fredrick, K., Cate, J.H. (2007). A 2teric block in translation caused by the antibiotic spectinomycin. Acs Chem.Biol. 2: 545-552 |
|  |  | 2QBB | 3.54 | Borovinskaya, M.A.,  Pai, R.D.,  Zhang, W.,  Schuwirth, B.S.,  Holton, J.M.,  Hirokawa, G.,  Kaji, H.,  Kaji, A.,  Cate, J.H., (2007) Nat.Struct.Mol.Biol. 14: 727-732 |
|  |  | 2QOY | 3.50 | Borovinskaya, M.A.,  Shoji, S.,  Holton, J.M.,  Fredrick, K.,  Cate, J.H., (2007) Acs Chem.Biol. 2: 545-552 |
|  |  | 1VS5 | 3.46 | Schuwirth, B.S.,  Day, J.M.,  Hau, C.W.,  Janssen, G.R.,  Dahlberg, A.E.,  Cate, J.H.D.,  Vila-Sanjurjo, A., (2006) Nat.Struct.Mol.Biol. 13: 879-886 |
|  | *Tetrahymena thermophila* | 2XZM | 3.93 | Rabl, J.,  Leibundgut, M.,  Ataide, S.F.,  Haag, A.,  Ban, N. (2011) Science **331:**730-736 |
| **Ribosomal Protein S5** | *Geobacillus stearothermophilus* | 1PKP | 2.80 | Ramakrishnan, V., White, S.W. (1992). The structure of ribosomal protein S5 reveals sites of interaction with 16S rRNA, Nature 358: 768-771. |
| **Ribosomal Protein S6** | *Aquifex aeolicus* | 2J5A | 2.30 | Olofsson, M., Hansson, S., Hedberg, L., Logan, D.T., Oliveberg, M. (2007). Folding of S6 structures with divergent amino acid composition: pathway flexibility within partly overlapping foldons, J.Mol.Biol. 365: 237 |
|  | *Thermotoga maritima* | 1VMB | 1.70 | Not available |
| **Ribosomal Protein S7** | *Geobacillus stearothermophilus* | 1HUS | 2.50 | Hosaka, H., Nakagawa, A., Tanaka, I., Harada, N., Sano, K., Kimura, M., Yao, M., Wakatsuki, S. (1997). Ribosomal protein S7: a new RNA-binding motif with structural similarities to a DNA architectural factor, Structure 5: 1199-1208 |
|  | *Pyrococcus horikoshii* | 1IQV | 2.10 | Hosaka, H., Yao, M., Kimura, M., Tanaka, I. (2001). The structure of the archaebacterial ribosomal protein S7 and its possible interaction with 16S rRNA, J.Biochem.(Tokyo) 130: 695-701 |
| **Ribosomal Protein S8** | *Geobacillus stearothermophilus* | 3RF2 | 2.16 | Menichelli, E., Edgcomb, S.P., Recht, M.I., Williamson, J.R. (2012). The structure of Aquifex aeolicus ribosomal protein S8 reveals a unique subdomain that contributes to an extremely tight association with 16S rRNA, J.Mol.Biol. 415: 489-502 |
| **Ribosomal Protein S15** | *Geobacillus stearothermophilus* | 1A32 | 2.10 | Clemons Jr., W.M., Davies, C., White, S.W., Ramakrishnan, V. (1998). Conformational variability of the N-terminal helix in the structure of ribosomal protein S15, Structure 6: 429-438 |
| **Ribosomal Protein S16** | *Aquifex aeolicus* | 3BN0 | 2.00 | Wallgren, M., Aden, J., Pylypenko, O., Mikaelsson, T., Johansson, L.B.-A., Rak, A., Wolf-Watz, M. (2008). Extreme temperature tolerance of a hyperthermophilic protein coupled to residual structure in the unfolded state, J.Mol.Biol. 379: 845-858 |
| **Ribosomal Protein S19** | *Pyrococcus abyssi* | 2V7F | 1.15 | Gregory, L.A., Aguissa-Toure, A.H., Pinaud, N., Legrand, P., Gleizes, P.E., Fribourg, S. (2007). Molecular basis of Diamond-Blackfan anemia: structure and function analysis of RPS19, Nucleic Acids Res. 35: 5913 |
