# Supplementary material for: A Comparison of Structural and Evolutionary Attributes of Escherichia coli and Thermus thermophilus Small Ribosomal Subunits: Signatures of Thermal Adaptation
Source: PLoS One. 2013 Aug 5;8(8):e69898. doi: 10.1371/journal.pone.0069898 (PMC3734280; doi:10.1371/journal.pone.0069898)
Supplement: File S2 — The detailed methods and corresponding references are included here. (DOC) [file pone.0069898.s014.doc]

| **A comparison of structural and evolutionary attributes of *Escherichia coli* and *Thermus thermophilus* small ribosomal subunits: signatures of thermal adaptation**  Saurav Mallik, and Sudip Kundu^*^  Department of Biophysics, Molecular Biology and Bioinformatics, University of Calcutta  Address: 92, APC Road, Kolkata; India; Zip: 700009; Email: Saurav Mallik: saurav.bmb@gmail.com; Sudip Kundu: skbmbg@caluniv.ac.in |
| --- |

**SUPPORTING INFORMATION-2**

**COMPUTATIONAL ANALYSIS**

The computational methods, programs, and web tools used are described in detail in the following.

**Alignment of 16S rRNAs**

We have structurally aligned the 16S rRNAs of *T. thermophilus* and *E. coli* to perform our cavity analysis. The alignment is performed using R3D Align program (Rahrig et al. 2010).

**Analysis of protein-rRNA and protein-protein interfaces**

The structural characterization of protein-protein and protein-RNA interfaces were first conducted by Janin *et al.* in 1988, Miller in 1989, Argos in 1988 and Thornton and coworkers (Jones and Thornton, 1995, 1996; Jones et al., 1999, 2001), which concentrated both on the protein-protein and protein-nucleic acid interfaces characterized in terms of hydrophobicity, accessible surface area, interface shape and residue propensities. The comparisons of different types of protein complexes (enzyme-inhibitor and antibody-antigen) in terms of interface size and hydrophobicity have been analyzed by Janin and coworkers (Janin and Chothia, 1990; Duquerroy et al., 1991). Korn and Burnett in 1991, Young et al. in 1994 and Thornton et and coworkers have contributed progressively on the protein-protein interfaces thereafter. The researches on protein-nucleic acid interfaces started with some pioneer works of Steitz in 1990, Harrison in 1991 and Thornton and coworkers (Jones et al., 1999; Luscombe et al., 2000; Jones et al., 2001), where they concentrated on the characterization of the corresponding interfaces. Olson and co-workers (Olson 19996; Olson and Zhurkin, 1996; Olson et al., 1998) detected the DNA structures distort upon protein binding which was also supported by Dickerson in 1998. These works also have revealed the sequence dependent nature of the protein-DNA binding sites. However, the protein-RNA interfaces have also been analyzed in detail, with the example of Thornton et al. in 2001.

In case of protein-protein contacts, an amino acid residue is defined as an interface residue if it loses >1Å^2^ of its accessible surface area when passing from uncomplexed state to complexed state (Jones et al., 1995). Jones et al. in 1999 and 2001 has shown that this is applicable to protein-RNA and protein-DNA complexes as well. By calculating the accessible surface area of the amino acid residues in the complexed three dimensional conformation both in complexed state (protein-RNA) and being isolated from the complex (only protein), it is possible to identify the amino acid residues with accessible surface area reduced >1Å^2^ on complex formation with ribosomal RNA. They are known as the interface residues and the total number of interface residues in a single protein defines its RNA binding site (Janin et al., 1998). The Surface Racer program (Tsodikov et al., 2002) was used to calculate the accessible and buried surface areas of the proteins and RNA, with probe radius taken to be 1.4Å, which resembles the radius of one water molecule. We calculated the water accessible surface area (ASA) of the two interacting partners using Surface racer program separately (in their complexed conformation) and in associated state. If the ASA of the two partners are A1 and A2 and of their associated structure is A3, then buried surface area is

$$BSA=\frac{1}{2}(A1+A2-A3)---------(1)$$

These results were compared with those provided by PDBePisa (Krissinel and Henrick, 2005, 2007; Krissinel 2009) web server of EBI (URL: http://ebi.ac.uk/msd-srv/prot-int/cgi-bin/piserver).

**The solvent free energy of interface formation, interface polarity and molecular contacts**

The extent of structural stability achieved by the interactions of two biomolecules can be predicted by the free energy (ΔG) change due to interaction. We predicted the solvent free energy of association using PDBePISA (Krissinel and Henrick, 2005, 2007; Krissinel 2009) web server of EBI (URL: http://ebi.ac.uk/msd-srv/prot-int/cgi-bin/piserver).

$\%polarity=\frac{BSA(polar)}{BSA(total)}\times100---------(2)$The %polarity of any protein-protein or protein-nucleic acid interface is defined as:

where BSA(polar) is the buried surface area of polar atoms and BSA(total) is the buried surface area of the total protein due to complex formation. For each ribosomal protein, this equation was used to calculate the %polarity of its interface with 16S rRNA.

**Analysis of molecular contacts**

We focused on the main two kinds of atom-atom contacts stabilizing the protein-rRNA interfaces: hydrogen bonds and van der Waals contacts. Intermolecular van der Waals contacts were calculated for protein-RNA complexes using NCONT program of ccp4i program suite (Winn et al., 2011), and Hydrogen bonds were calculated using Discovery Studio Visualizer program (Accelrys Software Inc.) and UCSF Chimera software (Pettersen et al., 2004). The theoretical criteria used to define a hydrogen bond was the D-A distance ≤3.35Å, and D-H-A angle to be within 90^0^-180^0^, where D stand for the donor group and A for the acceptor group. The van der Waals contacts between protein and RNA were defined as all contacts between atoms excluding those involved in hydrogen bonds that are ≤4.0Å apart (Jones et al., 2001). Distance criteria for van der Waals contact within protein are taken to be ≤5.0Å. For each ribosomal protein, the surface density of Hydrogen bonding, defined as the number of hydrogen bonds per 100Å^2^ buried surface area due to complex formation, was calculated from the available data, both at its RNA and protein-binding sites. All the data were compiled and presented according to the classifications adopted for the ribosomal proteins.

**Predicting structural flexibility of r-proteins prior their association in SSU and protein conformational changes due to SSU association**

$ASA=4.84M^{0.760}--------(3)$Binding of proteins with other proteins or nucleic acids is usually accompanied by significant changes of their three-dimensional conformations. There are prior works that predicted that there is a relationship between the conformational changes a protein undergoes upon complex formation and the interface size of the resulting complex. Janin et al. (1990) predicted that complexes with large interfaces must require major structural changes upon complex formation due to the excessive accessible surface of their isolated subunits. Lo Conte et al. (1999) showed that the subunits of protein complexes with large interfaces (BSA >2000Å^2^) tend to undergo greater conformational changes upon complex formation, compared to those with smaller interfaces. The connection between the intrinsic flexibility of unbound proteins and the conformations they adopt upon binding has been analyzed in detail by Marsh and Teichmann (2011). Analyzing accessible surface area of 4988 monomeric proteins Marsh and Teichmann were able to find out a relationship between molecular weight (M) and accessible surface area for all monomers.

$ASA(rel)=\frac{ASA(observed)}{ASA(predicted)}---------(4)$Since this equation is highly predictive of the ASA of monomeric and folded proteins, the authors concluded that the deviation of ASA from its predicted value might be a useful indicator of to what extent a protein resembles a folded monomer. They defined relative solvent accessible surface area, denoted by ASA(rel) as observed ASA scaled by its predicted value from the molecular weight:

$RMSD(\hom omer)=\exp\{6.14\times ASA(rel)^{complexed}-5.95\}----(5)$The ASA(rel) value for each subunit is to be considered in its bound conformation, isolated from the rest of the complex. By plotting the ASA(rel) with RMSD values of complexed and uncomplexed conformations, the authors have observed the following relationships:

$$RMSD(heteromer)=\exp\{6.35\times ASA(rel)^{complexed}-6.05\}----(6)$$

Applying this algorithm proposed by Marsh and Teichmann (2012) we have tried to predict the three dimensional conformational changes of all the r-proteins due to their transition from the complexed state to the uncomplexed state. Since ribosomal proteins are all heteromers, the second equation will tell us the RMSD values between the complexed and uncomplexed states of each ribosomal protein. The ASA of the ribosomal proteins in their complexed conformation isolated from ribosomal assembly were calculated using Surface Racer program (Tsodikov et al. 2002), and exact molecular weights were calculated using Discovery Studio Visualizer software. All the available data were compiled according to the classifications of r-proteins we adopted.

The Marsh and Teichmann algorithm we applied here describes a strategy to predict whether a protein structure is intrinsically disordered or not. The ASA(rel) value of one monomer in bound state being ≥1.2 indicates the protein is structurally flexible in its uncomplexed state, for which it experiences high conformational changes. On the other hand, ASA(rel) ≥ 1.4 indicates the protein is intrinsically disordered in its uncomplexed state.

**Cavity analysis and protein-rRNA interface complementarity**

Cavities are defects in biomolecule structures (Connolly 1986, Hubbard 1994). Water molecules generally occupy these cavities (Rashin 1986, Williams 1994). Generally, absence of cavities in a protein indicates a highly packed structure. The characterization of cavities at the interior and interfaces or proteins (Sonavane and Chakrabarti 2008) and at protein-RNA and protein-DNA interfaces (Sonavane and Chakrabarti 2009) has already been characterized. We have used the concept of cavities to understand the signatures of thermal adaptation in SSU on internal packing of 16S rRNA and r-proteins. We also have utilized protein-rRNA interface cavities to analyze their complementarity for the two species.

We have classified ribosomal cavities into three categories: (1) 16S rRNA interior cavities, (2) r-protein interior cavities and (3) protein-rRNA interface cavities. We used the CASTp (Computed Atlas of Surface Topography of proteins) server (Binkowski et al. 2003) using the probe radius of 1.4 Å. Only the cavities with volume >11.5 Å^3^ (probe volume having a radius 1.4 Å) were selected for analysis. We estimated two basic properties regarding cavity analysis: cavity sphericity and cavity index at the interfaces.

$Sphericity=\pi^{1/3}(6V)^{2/3}/A---------(7)$Cavity sphericity is a widely used concept. It determines how spherical the shape of the cavity is. It is the ratio of volume to surface of a cavity relative to that for a sphere with the same volume. If the volume of the cavity is V and surface area is A, then

$C_{i}=\frac{1}{ASA}\sum_{j=1}^{N} v_{j}-----------(8)$The cavity index (Ci) for a protein-protein of protein-rRNA interface is defined as:

Where ASA is the interface area and N is the number of total interface cavities, v_j_ is the volume of j-th cavity. Small values of cavity index indicate a high complementary interface.

**Prediction of Disordered and Ordered regions of r-proteins**

No universally accepted definition of protein disorder exists. The thermodynamic definition of disorder in a polypeptide chain is the "random coil" structural state. The random coil state can best be understood as the structural ensemble spanned by a given polypeptide in which all degrees of freedom are used within the conformational space. In this work, we have followed this thermodynamic definition. There are many recent reports of Intrinsically Disordered Proteins (IDPs). These are proteins or domains that, in their native state, are either completely disordered or contain large disordered regions. Protein disorder is important for understanding protein function as well as protein folding pathways (Plaxco and Gross 2001 and Verkhivker et al. 2003). IDPs are thought to become ordered only when bound to another molecule or owing to changes in the biochemical environment (Dunker et al. 2001, Dunker et al. 2002, and Uversky 2002).

The current view on disorder is that disordered proteins allow for more interaction partners and modification sites (Wright and Dyson 1999, Liu et al. 2002, and Tompa, 2002). Perhaps disordered proteins provide a simple solution to having large intermolecular interfaces while keeping smaller protein, genome and cell sizes (Gunasekaran et al. 2003). We have used three different definitions of disorder: (1) Russell/Linding definition of disorder using GlobPlot server (Linding et al. 2003) version 2.3, located at http://globplot.embl.de/; (2) Loops/Coil definition of disorder using disEMBL server (Linding et al. 2003) version 1.5, located at http://dis.embl.de/; (3) Hot Loops definition of disorder using disEMBL server (Linding et al. 2003) version 1.5.

Disorder is generally thought to be a property of protein sequence (Linding et al. 2003) and often disordered regions of proteins show a disorder-to-order transition as they associate with other binding partner. No universally accepted definition of disorder-to-order transition exists. To encounter this, we have concentrated on the thermodynamic definition of disorder: the thermodynamic definition of disorder in a polypeptide chain is the "random coil" structural state; the random coil state can best be understood as the structural ensemble spanned by a given polypeptide in which all degrees of freedom are used within the conformational space. **Thus, the number of utilized degrees of freedom of any particular residue can be used to measure its disordered/ordered state. Once the otherwise disordered residue interacts with any binding partner (physical contact), its dynamics becomes dependent on the dynamics of the binding partner. In other words, interaction with a binding partner reduces the utilized degrees of freedom of a disordered residue and the residue becomes more ordered compared to its uncomplexed state.** Thus, any residue, predicted to be disordered, if observed to be interacting with a binding partner in crystal structure, we have considered that the residue has experienced a **disorder-to-order transition** due to interaction.

On the other hand, if a residue predicted to be ordered, is observed to interact with a binding partner in crystal structure, we have considered that the residue has experienced an **order-to-order transition** due to interaction. In this transition, the residue experiences a transition from one ordered state to another ordered state.

**Evolutionary Analysis**

For the analysis of conservation at various sites of r-proteins, we have used the *Escherichia coli* and *Thermus thermophilus* r-protein sequences for PSI-BLAST (Altschul et al. 1997) against the known protein sequences available in Uniport database UniProt Consortium 2012). Resulting sequences were aligned using COBALT alignment tool (Papadopoulos and Agarwala 2007). This alignment was again verified using MUSCLE multiple alignment program (Edgar 2004). Scorecons program (Sedaghatinia et al. 2009) was used to predict conservation scores at the amino acid sites.

**Statistical Analysis: Data Clustering and Significance tests**

A number of physical properties are calculated and analyzed by statistical methods in this paper. Simple Hierarchical clustering algorithms (Gronau and Moran, 2007) were used in our analysis. Statistical significance tests were performed in terms of Mann-Whitney U-test (Mann and Whitney, 1947). The null hypothesis adopted: the two groups of data selected for analysis originate from the same population. The alternate hypothesis is: the two sets of data selected for analysis originate from two different populations. The null hypothesis is rejected and the alternate hypothesis is accepted when p≤0.01. When 0.01<p<0.05, we concluded that the two sets of data are marginally different (different populations). The two groups of data are considered to be originated from the same population, if p≥0.05.

All the clustering algorithms and U-tests were carried on using PAST statistical software package (Hammer et al., 2001). In this work, we also have tried to correlate some interface parameters, the correlation coefficients of whom were also calculated using PAST. The statistics of various linear and surface fitting were calculated using Origin data analysis and graphic workspace (OriginLab Corporation). Origin was also used to produce graph plots (Supporting Figure-3).

**REFERENCES**

Altschul SF, Madden TL, Schäffer AA, Zhang J, Zhang Z, et al. (1997) Gapped BLAST and PSI-BLAST: a new generation of protein database search programs, Nucleic Acids Res. 25(17): 3389-402.

Argos P (1988) An investigation of protein subunit and domain interfaces. Protein Eng. 2: 101-113.

Binkowski TA, Naghibzadeh S Liang J (2003) CASTp: computed atlas of surface topography of proteins. Nucleic Acids Res. 31: 3352–3355.

Connolly ML (1986) Atomic size packing defects in proteins. Int. J. Pept. Protein Res. 28: 360–363.

Crooks GE, Hon G, Chandonia J, Brenner SE (2004) WebLogo: a sequence logo generator. Genome Research 14: 1188–1190.

Dickerson RE (1998). DNA bending: the prevalence of kinkiness and the virtues of naormality. Nucleic Acids Research 26(8): 1906–1926.

Discovery Studio Visualizer, Copyright ©2010, Accelrys Software Inc. All rights reserved.

Dunker AK, Brown CJ, Lawson JD, Iakoucheva LM, Obradovic Z (2002) Intrinsic disorder and protein function. Biochemistry, 41: 6573-6582.

Dunker AK, Lawson JD, Brown CJ, Williams, RM, Romero P, et al. (2001) Intrinsically disordered protein. J. Mol. Graph Model. 26-59.

Duquerroy S, Cherfils J, Janin J (1991). Protein-protein interaction: an analysis by computer simulation. Ciba Found. Symp. 161: 237-252.

Edgar RC (2004) MUSCLE: multiple sequence alignment with high accuracy and high throughput, Nucleic Acids Research 32(5): 1792-97.

Gronau I, Moran S (2007) Optimal Implementations of UPGMA and Other Common Clustering Algorithms. Information Processing Letters, 104(6): 205-210.

Gunasekaran K, Tsai C, Kumar S, Zanuy D, Nussinov R (2003) Extended disordered proteins: targeting function with less scaffold. Trends Biochem. Sci. 28: 81-85.

Hammer Ø, Harper, DAT, Ryan PD (2001) PAST: Paleontological Statistics software package for education and data analysis. Paleontological Electronica 4(1): 9. Version 2.10 used.

Harrison SC (1991) A structural taxonomy of DNA-binding domains. Nature 353: 715-719.

Hubbard SJ, Gross KH, Argos P (1994) Intramolecular cavities in globular proteins. Prot. Eng. 7: 613–626.

Janin J, Miller S, Chothia C (1988) Surface, subunit interfaces and interior of oligomeric proteins. J. Mol. Biol. 204: 155–164.

Janin J Chothia C (1990) The structure of protein-protein recognition sites. J. Biol. Chem. 265: 16027-16030.

Jones S, Daley DTA, Luscombe NM, Berman HM, Thornton JM (2001) Protein-RNA interactions: a structural analysis. Nucl Acid Res. 29(4): 943-954.

Jones S, Heyningen PV, Berman HM, Thornton JM (1999) Protein-DNA interactions: a structural analysis. J. Mol. Biol. 287: 877-896.

Jones S, Thornton JM (1995) Protein-protein interactions: a review of protein dimer structures. Prog. Biophys. Mol. Biol. 63: 31-65.

Jones S, Thornton JM (1996) Principles of protein-protein interactions. Proc. Natl. Acad. Sci. USA. 93: 13-20.

Korn AP, Burnett RM (1991) Distribution and complementarity of hydropathy in multi-subunit proteins. Proteins: Struct. Funct. Genet. 9: 37-55.

Krissinel E (2009) Crystal contacts as nature's docking solutions. J Comput Chem. 31(1): 133-43.

Krissinel EB, Winn MD, Ballard CC, Ashton AW, Patel P, et al. (2004) The new CCP4 coordinate library as a toolkit for the design of coordinate related applications in protein crystallography. Acta Cryst. D60: 2250–2255.

Krissinel E, Henrick K (2004) Secondary structure matching (SSM), a new tool for fast protein structure alignment in three dimensions. Acta Cryst. D60: 2256-2268.

Krissinel E, Henrick K (2005) Detection of Protein Assemblies in Crystals. In: M.R. Berthold et.al. (Eds.): CompLife, LNBI 3695: 163--174. Springer-Verlag Berlin Heidelberg.

Krissinel E, Henrick K (2007) Inference of macromolecular assemblies from crystalline state. J. Mol. Biol. 372: 774--797.

Linding R, Jensen LJ, Diella F, Bork P, Gibson TJ, Russell RB (2003) Protein disorder prediction: implications for structural proteomics, Structure 11(11): 1453-9.

Linding R, Russell RB, Neduva V, Gibson TJ (2003) GlobPlot: Exploring protein sequences for globularity and disorder, Nucleic Acids Res. 31(13): 3701-8.

Liu J, Tan H, Rost B (2002). Loopy proteins appear conserved in evolution. J. Mol. Biol. 322: 53-64.

Lo Conte L, Chothia C, Janin J (1999) The atomic structure of protein-protein recognition sites. J. Mol. Biol. 285: 2177–2198.

Luscombe NM, Austin SE, Berman HM, Thornton JM (2000) An overview of the structures of protein-DNA complexes. Genome Biol 1: 1-37.

Mann HB, Whitney DR (1947) On a Test of Whether one of Two Random Variables is Stochastically Larger than the Other. Annals of Mathematical Statistics 18 (1): 50–60.

Marsh JA, Teichmann SA (2011) Protein solvent accessible surface area predicts protein conformational changes upon binding. Structure. 19(6): 859–867.

Miller S (1989) The structure of interfaces between subunits of dimeric and tetrameric proteins. Protein Eng. 3: 77-83.

Olson WK (1996) Simulating DNA at low resolution. Curr. Opin. Struct. Biol. 6: 242-256.

Olson WK, Zhurkin VB (1996) Twenty years of DNA bending. In Ninth Conversation in Biomolecular Stereodynamics, Adenine Press, Albany, NY.

Olson WK, Gorin AA, Lu XJ, Hock LM, Zhurkin VB (1998) DNA sequence-dependent deformability deduced from protein-DNA crystal complexes. Proc. Natl Acad. Sci. USA, 95: 11163-11168.

OriginPro 8 SR0, v8.0724 (B724). Copyright © 1991-2007 OriginLab Corporation.

Papadopoulos JS, Agarwala R (2007) COBALT: constraint-based alignment tool for multiple protein sequences, Bioinformatics 23(9): 1073-9.

Pettersen EF, Goddard TD, Huang CC, Couch GS, Greenblatt DM, Meng EC, Ferrin TE (2004) UCSF Chimera - A Visualization System for Exploratory Research and Analysis. J. Comput. Chem. 25: 1605-1612.

Plaxco KW, Gross M (2001) Unfolded, yes, but random? never! Nat. Struct. Biol. 8: 659-660.

Rahrig RR, Leontis NB, Zirbel CL (2010) R3D Align: global pairwise alignment of RNA 3D structures using local superpositions. Bioinformatics 26(21): 2689-97.

Rashin AA, Iofin M, Honig B (1986) Internal cavities and buried waters in globular proteins. Biochemistry 25: 3619–3625.

Sedaghatinia A, Atan RB, Arifin KT, Murad MABA (2009) Comparison and Evaluation of Multiple Sequence Alignment Tools In Bininformatics, IJCSNS International Journal of Computer Science and Network Security, 9(7).

Sonavane S, Chakrabarti P (2008) Cavities and atomic packing in protein structures and interfaces, PLoS Comput Biol. 4(9): e1000188.

Sonavane S, Chakrabarti P (2009) Cavities in protein-DNA and protein-RNA interfaces, Nucleic Acids Res. 37(14): 4613-20.

Steitz TA (1990) Structural studies of protein-nucleic acid interaction: the source of sequence specific binding. Q. Rev. Biophys. 23: 205-210.

Tompa P (2002). Intrinsically unstructured proteins. Trends Biochem. Sci. 27: 527-533.

Tsodikov OV, Record MT Jr., Sergeev YV (2002) A novel computer program for fast exact calculation of accessible and molecular surface areas and average surface curvature. J. Comput. Chem. 23: 600-609.

UniProt Consortium. (2012) Reorganizing the protein space at the Universal Protein Resource (UniProt), Nucleic Acids Res. 40(Database issue): D71-5.

Uversky VN (2002) Natively unfolded proteins: a point where biology waits for physics. Protein Sci. 11: 739-756.

Verkhivker GM, Bouzida D, Gehlhaar DK, Rejto PA, Freer ST, Rose PW (2003) Simulating disorder-order transitions in molecular recognition of unstructured proteins: where folding meets binding. Proc. Natl. Acad. Sci. U.S.A. 100: 5148-5153.

Williams MA, Goodfellow JM, Thornton JM (1994) Buried waters and internal cavities in monomeric proteins. Protein Sci., 3: 1224–1235.

Winn MD, Ballard CC, Cowtan KD, Dodson EJ, Emsley P, et al. (2011) Overview of the CCP4 suite and current developments. Acta.Cryst. D67: 235-242.

Wright PE, Dyson HJ (1999) Intrinsically unstructured proteins: Re-assessing the protein structure-function paradigm. J. Mol. Biol., 293: 321-331.

Young L, Jernigan RL, Covell DG (1994) A role for surface hydrophobicity in protein-protein recognition. Protein Sci. 3: 717-729.
